# Total Synthesis of (−)-Psathyrin A Enabled by Radical Cyclization

**DOI:** 10.1021/jacs.5c11534

**Published:** 2025-08-25

**Authors:** Weizhao Zhao, Reem Al-Ahmad, Mingji Dai

**Affiliations:** † Department of Chemistry, Emory University, Atlanta, Georgia 30322, United States; ‡ Department of Pharmacology and Chemical Biology, School of Medicine, Emory University, Atlanta, Georgia 30322, United States

## Abstract

We report herein an enantioselective total synthesis of (−)-psathyrin A, an antibacterial diterpene natural product possessing a unique 6/4/5/5 tetracyclic carbon skeleton and seven contiguous stereocenters, including three adjacent all-carbon quaternary centers. Our synthesis begins with commercially available 2-methyl-2-cyclopenten-1-one, which was subjected to an enantioselective copper/NHC-catalyzed conjugate addition, followed by trapping the resulting enolate with 1-bromo-2-butyne to set up the first two stereocenters, including one all-carbon quaternary center. A Suzuki–Miyaura cross coupling introduces an aromatic ring as the six-membered ring precursor, and a gold­(I)-catalyzed Conia-ene reaction constructs the 5/5-fused bicyclic ring system and the second all-carbon quaternary center. Following Birch reduction of the aromatic ring, hydrolysis, and double bond isomerization, a Baran reductive olefin coupling, namely, MHAT-initiated olefin-enone radical cyclization, was employed to construct the four-membered ring and establish the third all-carbon quaternary center. This enabling radical cyclization completed the tetracyclic carbon framework for subsequent peripheral decorations, achieving the first total synthesis of (−)-psathyrin A in 19 steps.

## Graphical abstract

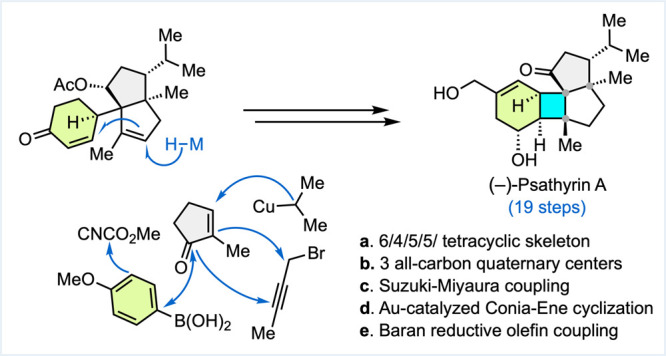

Psathyrins A (**1**, Figure 1A) and B (**2**) were isolated from the fermentation broth of *Psathyrella* *candolleana* by Liu, Feng, and co-workers in 2020.^1^ Structurally, the psathyrins represent two unique diterpene natural products featuring a complex and unprecedented 6/4/5/5 tetracyclic carbon skeleton. Each of them contains seven contiguous stereocenters, including three adjacent all-carbon quaternary centers, two of which reside on the strained cyclobutane ring. Biologically, psathyrins A and B were evaluated against Gram-positive *Staphylococcus aureus* and Gram-negative *Salmonella enterica* and *Pseudomonas aeruginosa*. Both compounds were found to exhibit weak activities against *Staphylococcus aureus* (MIC_50_ ∼ 14–23 μg/mL) and *Salmonella enterica* (MIC_50_ ∼ 78–102 μg/mL), but not *Pseudomonas aeruginosa* (MIC_50_ > 128 μg/mL).

**Figure 1:**
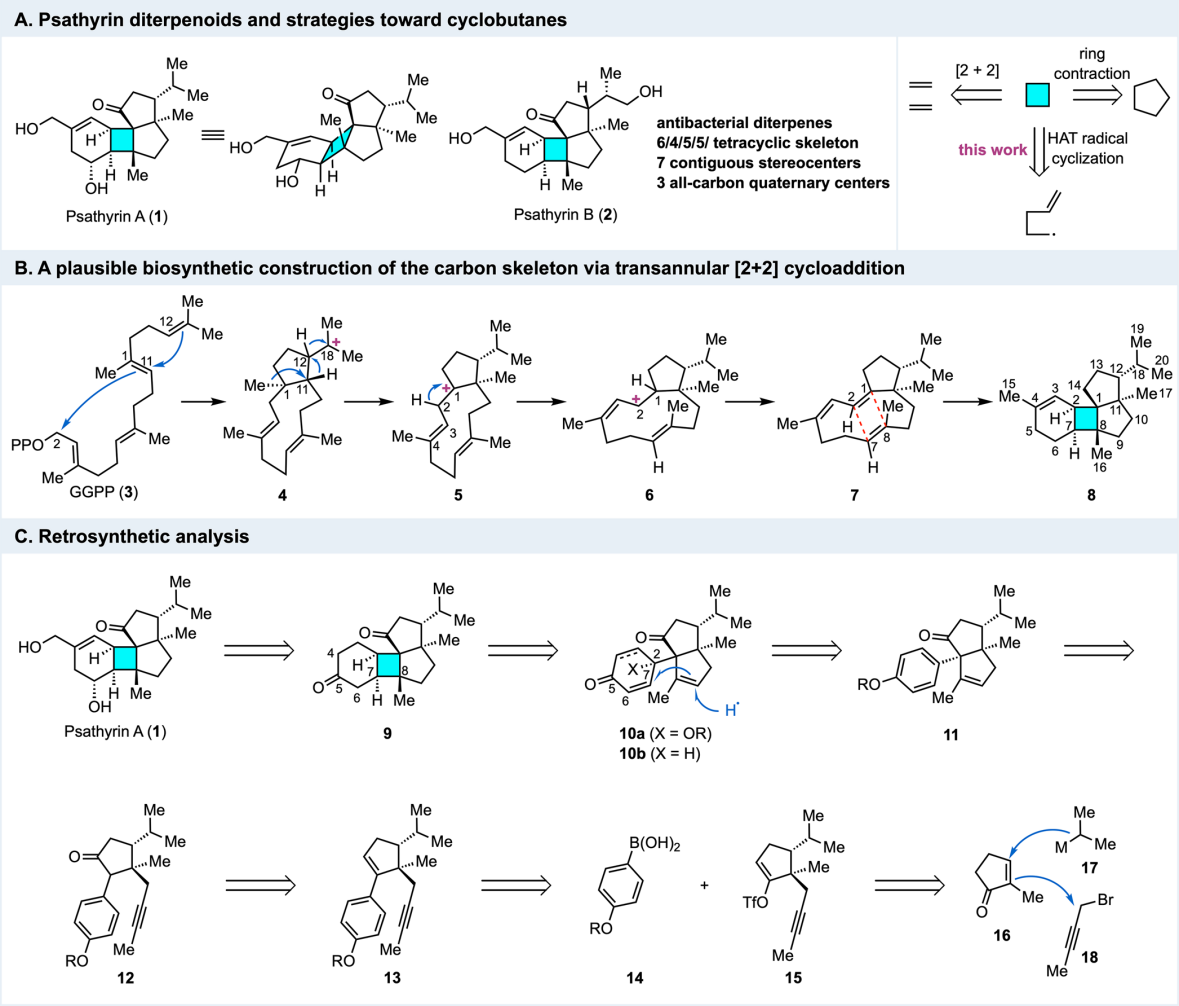
Structures, plausible biosynthesis, and retrosynthetic analysis.

Biosynthetically, psathyrins A and B were proposed to derive from geranylgeranyl pyrophosphate (GGPP, **3**, Figure 1B). A cationic polyene cyclization could form C1–C2 and C11–C12 bonds, generating the (*E*,*E*)-3,7-dolabelladiene cation (**4**). Subsequent hydride shifts (C12 to C18 and C11 to C12) and methyl shifts (C1 to C11) could generate a neodolabellane cation (**5**), from which Liu, Feng, and co-workers proposed two transannular cation-ene cyclizations and a series of hydride shifts to provide compound **8**, bearing the 6/4/5/5 tetracyclic carbon skeleton.^1^ Alternatively, we proposed another biosynthetic pathway from **5** to **8** involving a transannular [2 + 2] cycloaddition. Cation **5** could undergo a 1,2-hydride shift to give an allylic cation intermediate, which, after double bond isomerization, could give rise to allylic cation intermediate **6**, possessing a *Z* configuration at C3–C4. Loss of a proton from **6** could afford triene **7**. The latter (or its oxidized form) could then undergo a transannular [2 + 2] cycloaddition to deliver **8** (or its oxidized form). While there is no direct evidence to support this [2 + 2] biosynthetic pathway at this stage, such a process has been reported in the biosynthesis of several other cyclobutane-containing natural products.^2^

Our longstanding interest in the total synthesis of medicinally important terpene natural products^3^ and in leveraging metal-catalyzed hydrogen atom transfer (MHAT)-initiated radical cyclization for constructing strained ring systems^4^ prompted our pursuit of the psathyrins. These compounds feature a strained, highly substituted cyclobutane ring at the center of their tetracyclic framework. Such cyclobutane motifs pose a great synthetic challenge, and efficient approaches to their synthesis are highly desirable. Conventionally, cyclobutanes can be constructed via [2 + 2] cycloaddition reactions, such as photochemical [2 + 2] cycloadditions and ketene-alkene cycloadditions.^5^ Alternatively, ring contraction of a five-membered ring via reactions such as (Quasi-)­Favorskii rearrangement and Wolff rearrangement is also a common strategy for accessing cyclobutanes.^6^ In our total synthesis of peyssonnoside A, we utilized a counterintuitive MHAT-initiated radical cyclization to build its highly congested cyclopropane ring.^4^ The success of this work encouraged us to explore more MHAT-initiated radical cyclizations to build strained ring systems^7^ particularly those embedded in complex natural product skeletons. In this context, we chose psathyrin A as our target molecule and focused on investigating a MHAT-initiated radical cyclization strategy^8^ to build its highly congested and substituted cyclobutane ring instead of other strategies such as ring contraction and [2 + 2] cycloaddition.

Retrosynthetically (Figure 1C), compound **9** with a C5 ketone was designed as an advanced intermediate toward psathyrin A via late-stage peripheral decorations, including oxidation state adjustment and introduction of a hydroxymethyl group at C4. While C5 is a methylene as encoded by psathyrin A, it is strategically important to position a ketone functionality here to activate the C6–C7 olefin as a better radical acceptor for the proposed radical cyclization to forge the challenging C7–C8 bond. We envisioned two possible pathways for this radical cyclization: one involving dienone **10a** (X = OR), which could be prepared from an oxidative dearomatization of **11**, and the other using enone **10b** (X = H), which could be derived from **11** via a sequence of Birch reduction, hydrolysis, and double bond isomerization. For the former (**10a**), the stereochemical outcome at C2 is not a concern due to its symmetry; however, an additional alkoxy group at C2 and an extra double bond need to be removed at a later stage. For the latter (**10b**), it could lead to **9** directly, but controlling the stereochemistry at C2 was uncertain at the planning stage. For both **10a** and **10b**, we anticipated that the cyclization would give the desired stereochemistry at C7 and C8 so the 6/4/5 tricycle could take a chairlike shape to avoid steric repulsions. To access **11**, we proposed a gold­(I)-catalyzed Conia-ene reaction of **12** to form the desired five-membered ring and an all-carbon quaternary center.^9^ Compound **12** could be derived from **13**, which could be assembled by a Suzuki–Miyaura cross coupling between boronic acid **14** and vinyltriflate **15**. The latter could be traced back to 2-methyl-2-cyclopenten-1-one (**16**), 1-bromo-2-butyne (**18**), and an isopropyl nucleophile (**17**) via a sequence of enantioselective conjugate addition and enolate propargylation.^10^

Our synthesis started with **16** being converted to **20** via a tandem copper-catalyzed enantioselective conjugate addition of an isopropyl Grignard reagent and propargylation with **18** (Scheme 1). The enantiomer of **20** was prepared in our peyssonnoside A total synthesis.^4^ In the current case, the use of chiral *N*-heterocyclic carbene (NHC) ligand **19** gave product **20** in 87% yield and 81% ee on multigram scale. After **20** was converted to vinyltriflate **15** using the Comins’ protocol,^11^ a Suzuki–Miyaura cross coupling reaction^12^ between **15** and commercially available (4-methoxyphenyl)­boronic acid (**21**) gave **22** in good yield. Electrophilic epoxidation with *m*-CPBA gave epoxide **23**. Interestingly, **23** started to undergo a Meinwald rearrangement^13^ to afford ketone **24** in deuterated chloroform during NMR analysis. To further accelerate the rearrangement process, *p*-TsOH was added to the same reaction pot to provide ketone **24** in 87% yield from **22**. Notably, for the subsequent gold­(I)-catalyzed Conia-ene cyclization, the formation of silyl enol ether was not necessary presumably due to the acidity of the α-proton at C1. Upon treatment of **24** with a combination of AuCl­(PPh_3_) and AgOTf in toluene, bicyclic product **25** with vicinal all-carbon quaternary centers at its ring junction was produced in 81% yield to set up the stage for the proposed MHAT-initiated radical cyclization to form the four-membered ring and the third all-carbon quaternary center. Unfortunately, we were unable to prepare substrates such as **10a** via oxidative dearomatization protocols because the C14 ketone further reacted with the newly formed dienone to complicate the reaction. Our radical cyclization attempts with substrate **25** were unsuccessful as well. We then switched to preparing enone substrate **27**. The ketone functionality of **25** was first reduced to a secondary alcohol with LiAlH_4_. The subsequent one-pot Birch reduction and hydrolysis, followed by acetate protection of the secondary alcohol, gave **26** smoothly. The next double bond isomerization turned out to be nontrivial. The isomerization has low conversion and the diastereoselectivity is sensitive to the reaction time, temperature, and scale. When 1.79 g of **26** was treated with HCl (1.0 M in Et_2_O) in THF at 0 °C for 6 h, ∼ 30% conversion was achieved.^14^ The recovered starting material was subjected to the same reaction conditions again. After two cycles, product **27** was produced in 61% yield (dr = 8.5/1) with the rest of **26** recovered to avoid significant loss of the starting material. To our delight, the major diastereomer has the desired stereochemistry at C2. With the Baran reductive olefin coupling conditions (Fe­(acac)_3_, PhSiH_3_, EtOH),^7a^ compound **27** underwent MHAT-initiated radical cyclization to provide **28** in excellent yield, showcasing the efficiency of such MHAT chemistry in building strained ring systems!

**Scheme 1:**
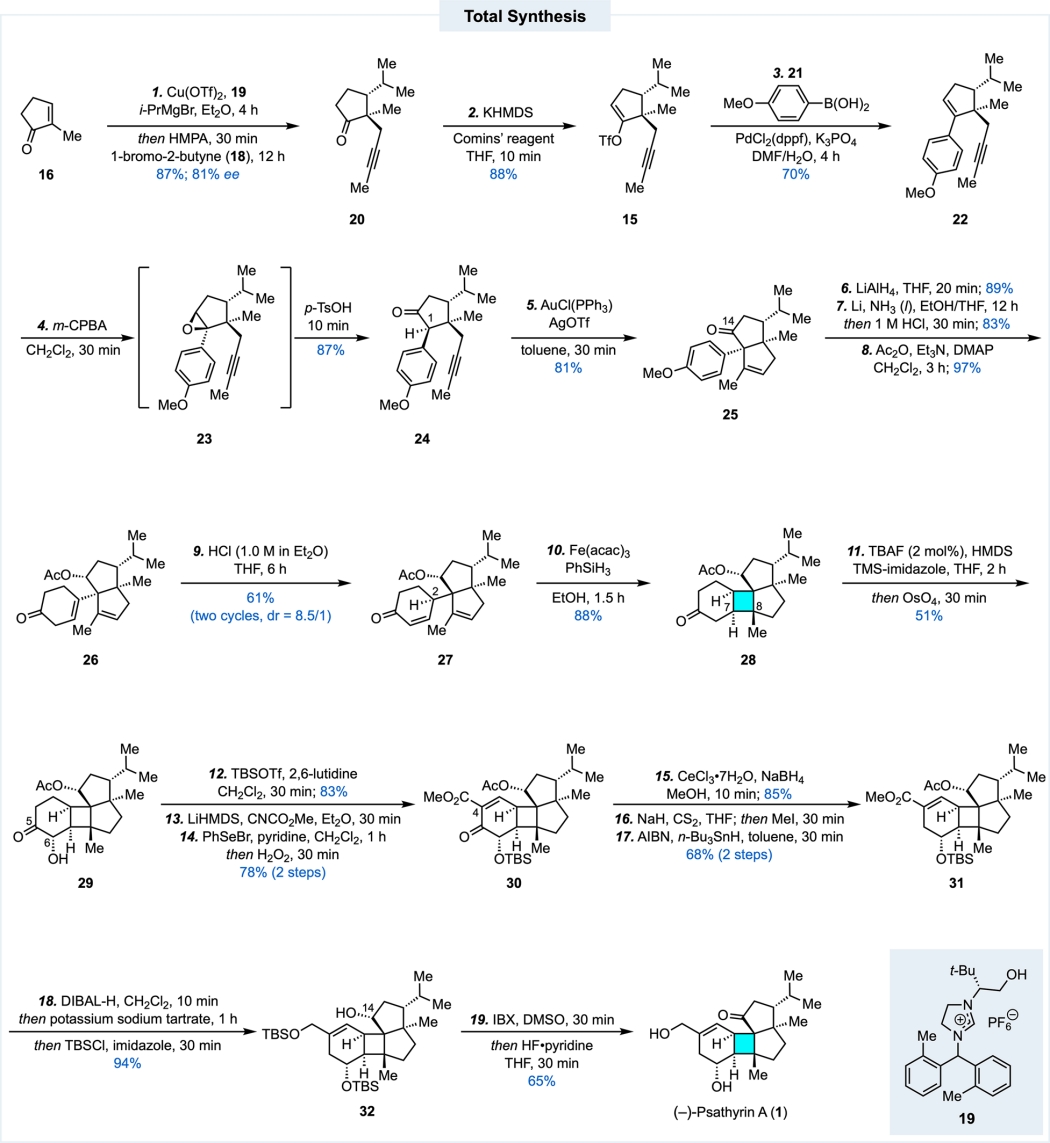
Total Synthesis of (−)-Psathyrin A

With tetracyclic product **28** in hand, we next focused on peripheral decorations to complete the total synthesis of (−)-psathyrin A. In addition to facilitating the MHAT-initiated radical cyclization, the C5 ketone functionality allowed introduction of a hydroxyl group at C6 via a one-pot Rubottom oxidation,^15^ namely, selective silyl enol ether formation and dihydroxylation, to give **29**. The resulting secondary alcohol was protected as a TBS ether. The C5 ketone further enabled us to introduce a methyl carboxylate group at C4 using Mander’s protocol.^16^ The carboxylate group served as a precursor of the hydroxymethyl group encoded by the target molecule. A subsequent one-pot α-selenation and oxidative *syn*-elimination afforded compound **30** in good yield. At this stage, it was time to reduce the C5 ketone to a methylene group and convert **30** to **31**. This task was achieved by a sequence of Luche reduction^17^ and Barton-McCombie deoxygenation.^18^ Notably, the strained cyclobutane ring survived this radical deoxygenation process. The carboxylate group and acetate group of **31** were then reduced with DIBAL-H. The resulting primary alcohol was selectively protected as a TBS ether to give **32** in 94% yield. IBX oxidation of the secondary alcohol at C4 followed by removal of the two TBS protecting groups with HF/pyridine completed the total synthesis of (−)-psathyrin A in 19 steps. Notably, while the synthetic sequence to advance the radical cyclization product **28** to (−)-psathyrin A (**1**) requires nine steps, the entire sequence from **28** to **1** could be completed in just 2 days due to the short reaction time and high efficiency of each step.

In summary, the first enantioselective total synthesis of (−)-psathyrin A was completed in 19 steps. Our synthesis centers on a MHAT-initiated radical cyclization to construct the highly congested and connected cyclobutane ring encoded by psathyrin A. This work not only showcases the efficiency and power of such MHAT chemistry in C–C bond formations but also broadens its applications to build strained ring systems. Other notable steps include a tandem enantioselective conjugate addition and alkylation to form the first two guiding stereocenters in the entire total synthesis, a Suzuki–Miyaura reaction to introduce an aromatic ring as the six-membered ring precursor, and a gold­(I)-catalyzed Conia-ene reaction to form the 5/5-fused bicyclic ring system and an all-carbon quaternary center. The application of radical cyclization strategies to synthesize strained molecules, particularly complex natural products, is currently ongoing in our laboratory and will be reported in due course.

## Supplementary Material



## Notes

Experimental procedures and NMR spectra for all new compounds (PDF)

## Abbreviations

HMPA: hexamethylphosphoramide
KHMDS: potassium bis­(trimethylsilyl)­amide
THF: tetrahydrofuran
DMF: dimethylformamide
*m*-CPBA: *meta*-chloroperoxybenzoic acid
DMAP: 4-dimethylaminopyridine
TBAF: tetra-*n*-butylammonium fluoride
HMDS: hexamethyldisilazane
TMS: trimethylsilyl
TBSOTf: *tert*-butyldimethylsilyl trifluoromethanesulfonate
LiHMDS: lithium bis­(trimethylsilyl)­amide
AIBN: 2,2′-azobis­(2-methylpropionitrile)
DIBAL-H: diisobutylaluminum hydride
TBSCl: *tert*-butyldimethylsilyl chloride
IBX: 2-iodoxybenzoic acid
DMSO: dimethyl sulfoxide
