# Supplementary material for: Total Synthesis of (−)-Psathyrin A Enabled by Radical Cyclization
Source: J Am Chem Soc. 2025 Aug 25;147(36):32365–9. doi: 10.1021/jacs.5c11534 (PMC12426916; doi:10.1021/jacs.5c11534)

Supporting Information

Total Synthesis of (−)-Psathyrin A Enabled by Radical Cyclization

Weizhao Zhao,[1] Reem Al-Ahmad,[1] and Mingji Dai*,[1,2]

[1]Department of Chemistry, Emory University, Atlanta, Georgia 30322, United States

[2]Department of Pharmacology and Chemical Biology, School of Medicine, Emory University, Atlanta, Georgia 30322, United States

Table of Contents

Part 1. Experimental Procedures and Spectra Data...S3
Part 2. Natural Product NMR Comparison..S21
Part 3. References..S24
Part 4. ^{1}H and ^{13}C NMR Spectra...S25

Part 1. Experimental Procedures and Spectra Data

A. General Methods

All commercially available compounds were purchased from Sigma-Aldrich, Alfa-Aesar, Oakwood chemicals and Ambeed unless otherwise noted. Materials obtained from commercial suppliers were used without further purification. NMR spectra were recorded on Bruker spectrometers (^{1}H at 400 MHz, 600 MHz, 800 MHz and ^{13}C at 101 MHz, 151 MHz, 201 MHz). Chemical shifts (δ) were given in ppm with reference to solvent signals [$CDCl_3$ δ 7.26 ppm ^{1}H NMR, δ 77.16 ppm ^{13}C NMR); CD_3OD, δ 3.31 ppm ^{1}H NMR, δ 49.0 ppm ^{13}C NMR]. ^{1}H NMR data are reported as follows: chemical shift (δ ppm), multiplicity (s = singlet, d = doublet, t = triplet, q = quartet, m = multiplet, br = broad), coupling constant (Hz), and integration. IR spectra were collected on a Nicolet iS10 FT-IR spectrometer. Mass spectra were taken on a Thermo Finnigan LTQ-FTMS spectrometer with APCI, ESI. Optical rotations were determined by Autopol IV (Rudolph Research Analytical). Column chromatography was performed on silica gel. All reactions sensitive to air or moisture were conducted under argon atmosphere in dry solvents under anhydrous conditions, unless otherwise noted. Dry THF (tetrahydrofuran), DMF (dimethylformamide), DCM (CH_2Cl_2) were processed via Pure Process Technology GS-SPS-5-CM system. All other solvents and reagents were used as obtained from commercial sources without further purification. Room temperature (r.t.) is around 23 °C.

B. Experiment Procedure and Spectra Data

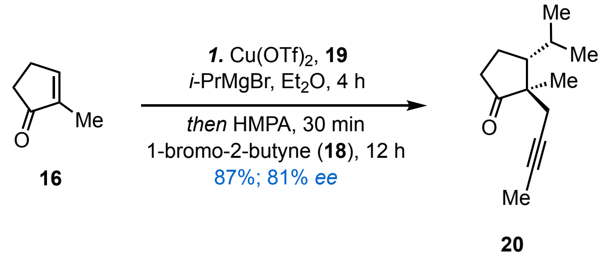

A flame-dried flask was charged with NHC Ligand **19** [1] (531 mg, 1.04 mmol, 2.0 mol%) and copper triflate (314 mg, 0.87 mmol, 1.67 mol%). Anhydrous diethyl ether (347 mL) was added, and the mixture was stirred for 10 min at room temperature, followed by 10 min at −30 °C. Isopropylmagnesium bromide **18** (21.54 mL, 62.46 mmol, 1.2 equiv.) was slowly added and the mixture was stirred for 30 min at −30 °C. 2-Methyl-

2-cyclopenten-1-one (5.0 g, 52.05 mmol, 1.0 equiv.) dissolved in anhydrous diethyl ether (50 mL) was slowly added with a syringe pump (along the inner wall of the flask over 2 h) at −30 °C. The crude mixture was allowed to react for 4 h and HMPA (87 mL, 520.05 mmol 10 equiv.) was then added at −30 °C. The reaction mixture was stirred for 30 min at room temperature before 1-Bromo-2-butyne (9.1 mL, 104.1 mmol, 2.0 equiv) was added dropwise at −30 °C. The reaction mixture was allowed to warm up to room temperature and stirred overnight. The reaction was quenched with saturated aqueous NH_4Cl (80 mL), and the resulting mixture was extracted with EtOAc (100 mL × 3). The combined organic phase was washed with brine (150 mL), dried over Na_2SO_4, filtered and concentrated under reduced pressure. The crude residue was purified by flash chromatography (2 to10% ethyl acetate in hexanes) to afford **20** (8.7 g, 87%, 81% ee) as a pale-yellow oil.

$\mathbf{R}_{\boldsymbol{f}}$ = 0.3 (hexane/ethyl acetate = 10/1).

$[\alpha]_D^{24}$ = −91.8 (*c* = 0.5 in MeOH).

IR (film) λ_{max}: 2963, 2955, 2920, 2876, 1739, 1473, 1458, 1372, 1070, 968, 772 cm^{-1}.

^{1}H NMR (400 MHz, $CDCl_3$) δ 2.56 (dq, *J* = 16.4, 2.6 Hz, 1H), 2.40 – 2.29 (m, 1H), 2.21 (dq, *J* = 16.4, 2.4 Hz, 1H), 2.16 – 1.96 (m, 3H), 1.73 (t, *J* = 2.6 Hz, 3H), 1.70 – 1.57 (m, 1H), 1.49 – 1.30 (m, 1H), 1.01 (d, *J* = 6.6 Hz, 3H), 0.97 (d, *J* = 6.6 Hz, 3H), 0.86 (s, 3H).

^{13}C NMR (101 MHz, $CDCl_3$) δ 223.0, 78.2, 75.9, 51.7, 48.8, 37.7, 29.5, 27.9, 24.3, 22.2, 21.7, 17.3, 3.6.

HRMS m/z (APCI): calc. for $C_{13}H_{21}O^+$ $[M+H]^+$: 193.1587, found: 193.1587.

HPLC Chromatogram: Racemic

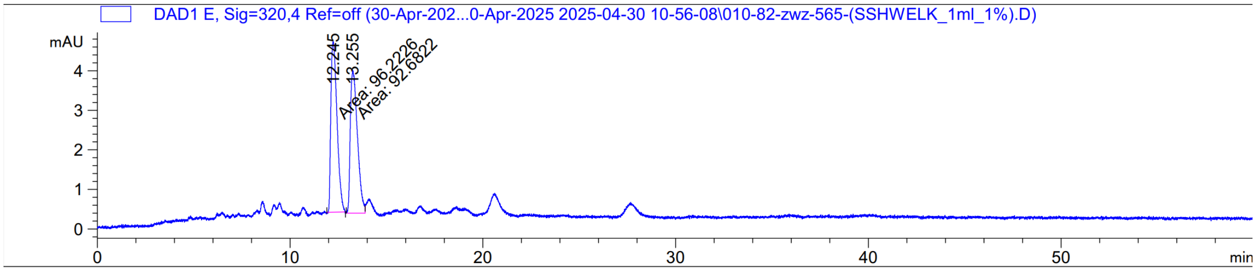

Peak #	RetTime [min]	Type	Width [min]	Area [mAU*s]	Height [mAU]	Area %
1	12.245	MM	0.3723	96.22256	4.30719	50.9371
2	13.255	MM	0.4273	92.68216	3.61461	49.0629
Totals :				188.90472	7.92181	

HPLC Chromatogram: Chiral

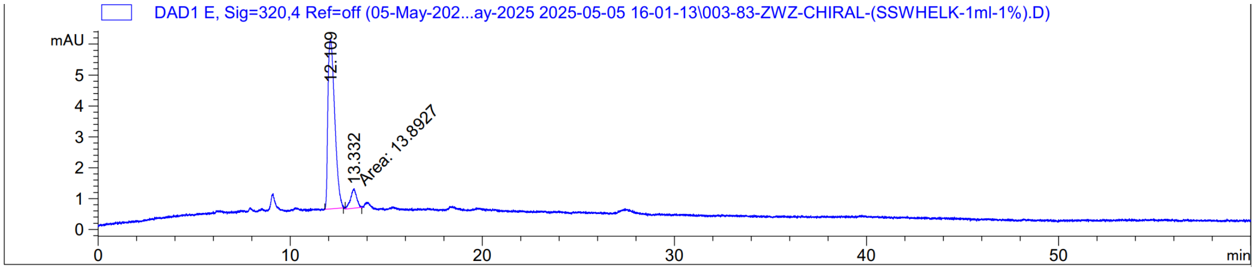

Signal 5: DAD1 E, Sig=320,4 Ref=off

Peak #	RetTime [min]	Type	Width [min]	Area [mAU*s]	Height [mAU]	Area %
1	12.109	BB	0.2886	134.47350	5.46471	90.6362
2	13.332	MM	0.3665	13.89271	6.31698e-1	9.3638
Totals :				148.36620	6.09640	

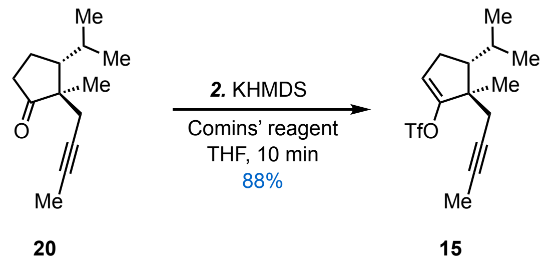

To a mixture of **20** (8.66 g, 45.50 mmol, 1.0 equiv.) and Comins' reagent (21.44 g, 54.6 mmol, 1.2 equiv.) in THF (182 mL) was added KHMDS (1.0 M in THF, 68.26 mL, 68.26 mmol, 1.5 equiv.) at −78 °C. The reaction mixture was stirred for 10 min at −78 °C. Upon complete consumption of starting material as indicated by TLC, the reaction was quenched with saturated aqueous $NaHCO_3$ (80 mL). The resultant mixture was extracted with EtOAc (60 mL × 3). The combined organic phase was washed with brine (100 mL), dried over anhydrous Na_2SO_4, filtered and concentrated under reduced pressure. The crude residue was purified by flash chromatography (2 to 5% ethyl acetate in hexanes) to afford **15** (12.98 g, 88%) as a pale-yellow oil.

R_f = 0.4 (hexane/ethyl acetate = 10/1).

$[\alpha]_D^{24}$ = −11.6 (c = 0.5 in MeOH).

IR (film) λmax: 2963, 2938, 2925, 2875, 2853, 1420, 1248, 1206, 1139, 1084, 765, 669 cm^{-1}.

^{1}H NMR (400 MHz, CDCl3) δ 5.57 (dd, J = 3.2, 2.0 Hz, 1H), 2.50 – 2.37 (m, 1H), 2.38 – 2.24 (m, 2H), 2.20 – 2.09 (m, 1H), 2.01 (ddd, J = 15.3, 9.0, 2.0 Hz, 1H), 1.78 (t, J = 2.6 Hz, 3H), 1.76 – 1.64 (m, 1H), 1.06 (s, 3H), 0.98 (d, J = 6.6 Hz, 3H), 0.91 (d, J = 6.6 Hz, 3H).

^{13}C NMR (101 MHz, CDCl3) δ 153.3, 118.7 (q, J = 320.1 Hz), 112.3, 78.9, 75.3, 49.2, 48.9, 31.3, 29.3, 28.4, 22.5, 21.6, 18.1, 3.7.

HRMS m/z (APCI): calc. for $C_{14}H_{20}O_3F_3S^+$ $[M+H]^+$: 325.1080, found: 325.1074.

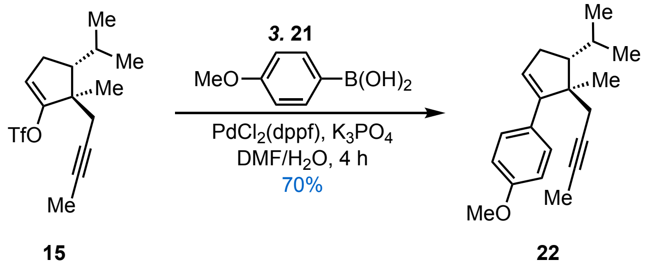

To a mixture of **15** (8.11 g, 25.02 mmol, 1.0 equiv.) and 4-methoxyphenylboronic acid (**21**, 5.7 g, 37.53 mmol, 1.5 equiv.) in DMF/H_2O (150 mL/15 mL) was added K_3PO_4 (10.62 g, 54.05 mmol, 2.0 equiv.) and $PdCl_2$(dppf) (3.69 g, 5.01 mmol, 0.2 equiv.) at room temperature. The reaction mixture was stirred for 4 h at room temperature. Upon complete consumption of starting material as indicated by TLC, the reaction was quenched with saturated aqueous $NaHCO_3$ (200 mL). The resultant mixture was extracted with EtOAc (3 × 80 mL). The combined organic phase was washed with brine (150 mL), dried over anhydrous Na_2SO_4, filtered and concentrated under reduced pressure. The crude residue was purified by flash chromatography (2 to 10% ethyl acetate in hexanes) to afford **22** (4.94 g, 70%) as a pale-yellow oil.

R_f = 0.3 (hexane/ethyl acetate = 10/1).
$[\alpha]_D^{24}$ = −75.2 (c = 0.5 in MeOH).
IR (film) λ_{max}: 2956, 2933, 2928, 2919, 2872, 2835, 1607, 1510, 1464, 1248, 1178, 833, 694 cm^{-1}.
^{1}H NMR (400 MHz, $CDCl_3$) δ 7.24 (d, J = 8.8 Hz, 2H), 6.84 (d, J = 8.8 Hz, 2H), 5.60 (t, J = 2.4 Hz, 1H), 3.81 (s, 3H), 2.46 (ddd, J = 15.8, 7.8, 2.8 Hz, 1H), 2.37 – 2.22 (m, 3H), 2.06 (ddd, J = 16.0, 9.2, 2.0 Hz, 1H), 1.84 – 1.71 (m, 4H, overlap), 1.03 (d, J = 6.6 Hz, 3H), 1.01 (s, 3H), 0.95 (d, J = 6.6 Hz, 3H).
^{13}C NMR (101 MHz, $CDCl_3$) δ 158.6, 151.2, 130.8, 129.8, 127.5, 113.3, 78.2, 77.8, 55.3, 52.0, 52.0, 35.0, 29.6, 29.4, 22.5, 22.4, 19.8, 3.9.
HRMS m/z (APCI): calc. for $C_{20}H_{27}O^+$ $[M+H]^+$: 283.2056, found: 283.2056.

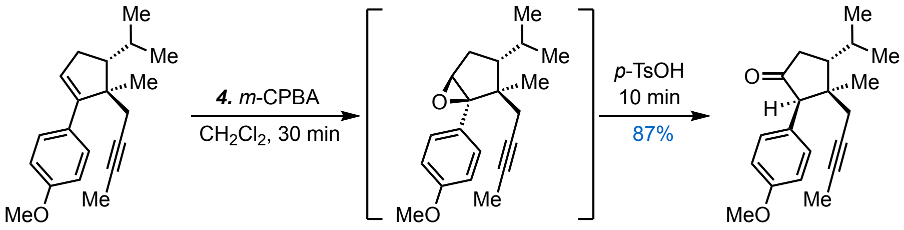

22 **23** **24**

To a solution of **22** (4.8 g, 17.0 mmol, 1.0 equiv.) in DCM (170 mL) was slowly added m-CPBA (75%, 7.82 g, 34.0 mmol, 2.0 equiv) at 0 °C. The reaction mixture was stirred at 0 °C for 30 min. After TLC showed full consumption of starting material p-TsOH·H_2O (6.47 g, 34.0 mmol, 2.0 equiv) was added. The reaction mixture was stirred for an additional 10 min at 0 °C before it was carefully quenched with saturated aqueous $NaHCO_3$ (120 mL). The resultant mixture was extracted with DCM (3 × 100 mL). The combined organic phase was washed with brine (200 mL), dried over anhydrous Na_2SO_4, filtered and concentrated under

reduced pressure. The crude residue was purified by flash chromatography (10 to 20% ethyl acetate in hexanes) to afford **24** (4.41 g, 87%) as a colorless oil.

Note: *m*-CPBA is explosive and should be used with caution!

R_f = 0.3 (hexane/ethyl acetate = 5/1).

$[\alpha]_D^{24}$ = −53.2 (c = 0.2 in MeOH).

IR (film) λ_{max}: 2956, 1738, 1514, 1465, 1380, 1251, 1181, 1033, 749, 661 cm^{-1}.

^{1}H NMR (400 MHz, $CDCl_3$) δ 7.02 (d, J = 8.7 Hz, 2H), 6.85 (d, J = 8.7 Hz, 2H), 3.79 (s, 3H), 3.44 (d, J = 1.5 Hz, 1H), 2.61 (dd, J = 19.5, 9.6 Hz, 1H), 2.43 – 2.28 (m, 2H), 2.12 (pd, J = 6.7, 3.6 Hz, 1H), 2.00 – 1.89 (m, 1H), 1.79 – 1.67 (m, 4H, overlap), 1.21 (s, 3H), 0.99 (d, J = 6.8 Hz, 3H), 0.96 (d, J = 6.8 Hz, 3H).

^{13}C NMR (101 MHz, $CDCl_3$) δ 218.5, 158.8, 131.4, 127.2, 113.8, 78.7, 77.0, 64.9, 55.3, 46.9, 45.6, 38.5, 29.4, 28.4, 23.6, 21.6, 19.0, 3.6.

HRMS m/z (APCI): calc. for $C_{20}H_{27}O_2^+$ $[M+H]^+$: 299.2006, found: 299.1997.

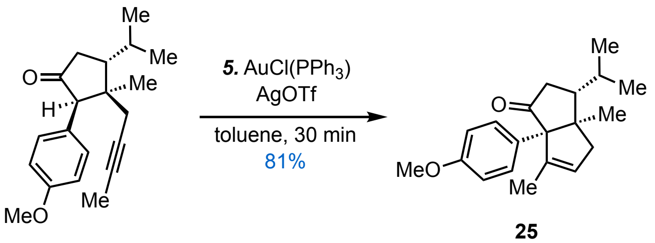

24 **25**

To a mixture of $AuCl(PPh_3)$ (1.46 g, 2.95 mmol, 0.2 equiv.) and AgOTf (0.76 g, 2.95 mmol, 0.2 equiv.) in toluene (148 mL) was added **24** (4.4 g, 14.76 mmol, 1.0 equiv.) at room temperature. The reaction mixture was heated to 80 °C and stirred at this temperature for 30 min. Upon complete consumption of starting material as indicated by TLC, the reaction was quenched with saturated aqueous $NaHCO_3$ (80 mL). The resultant mixture was extracted with EtOAc (3 × 100 mL). The combined organic phase was washed with brine (100 mL), dried over anhydrous Na_2SO_4, filtered and concentrated under reduced pressure. The crude residue was purified by flash chromatography (8 to 15% ethyl acetate in hexanes) to afford **25** (3.57 g, 81%) as a colorless oil.

R_f = 0.35 (hexane/ethyl acetate = 5/1).

$[\alpha]_D^{24}$ = −21.5 (c = 0.2 in MeOH).

IR (film) λ_{max}: 3034, 2961, 2914, 2894, 2871, 2837, 1727, 1608, 1510, 1465, 1250, 1180, 822, 752 cm^{-1}.

^{1}H NMR (400 MHz, $CDCl_3$) δ 6.88 – 6.79 (m, 4H), 5.71 – 5.63 (m, 1H), 3.78 (s, 3H), 2.64 – 2.48 (m, 2H), 2.20 – 2.07 (m, 2H), 1.85 – 1.62 (m, 2H), 1.61 – 1.54 (m, 3H), 1.05 (d, J = 6.4 Hz, 3H), 0.92 (d, J = 6.4 Hz, 3H), 0.60 (s, 3H).

^{13}C NMR (101 MHz, $CDCl_3$) δ 218.3, 158.4, 140.4, 130.3, 129.3, 128.7, 113.8, 79.0, 55.3, 54.9, 49.2, 45.1, 44.8, 30.4, 23.3, 22.9, 17.9, 13.9.

HRMS m/z (APCI): calc. for $C_{20}H_{27}O_2^+$ $[M+H]^+$: 299.2006, found: 299.2005.

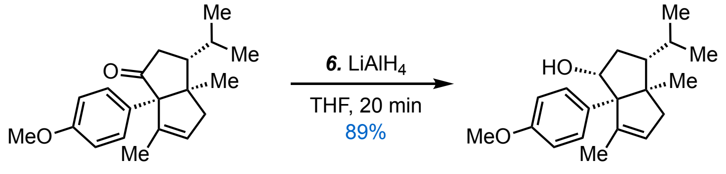

25 **SI-1**

To a solution of **25** (3.25 g, 10.9 mmol, 1.0 equiv.) in THF (109 mL) was carefully added $LiAlH_4$ (827 mg, 21.8 mmol, 2.0 equiv.) at 0 °C. The reaction mixture was stirred for 20 min at 0 °C before it was poured into an Erlenmeyer flask containing saturated aqueous $NaHCO_3$ (100 mL). The resulting mixture was extracted with EtOAc (3 × 80 mL). The combined organic phase was washed with brine (100 mL), dried over anhydrous Na_2SO_4, filtered and concentrated under reduced pressure. The crude residue was purified by flash chromatography (8 to 20% ethyl acetate in hexanes) to afford **SI-1** (2.91 g, 89%) as a pale-yellow oil.

R_f = 0.3 (hexane/ethyl acetate = 5/1).

$[\alpha]_D^{24}$ = +24.0 (c = 0.2 in MeOH).

IR (film) λ_{max}: 3428, 3034, 2956, 2870, 2840, 1609, 1510, 1456, 1248, 1182, 824, 697 cm^{-1}.

^{1}H NMR (400 MHz, $CDCl_3$) δ 7.11 (d, J = 8.8 Hz, 2H), 6.87 (d, J = 8.8 Hz, 2H), 5.42 – 5.35 (m, 1H), 4.12 (dd, J = 11.6, 6.2 Hz, 1H), 3.80 (s, 3H), 2.42 – 2.35 (m, 1H), 2.28 – 2.19 (m, 1H), 2.00 – 1.89 (m, 1H), 1.70 – 1.74 (m, 3H), 1.69 – 1.58 (m, 3H), 1.43 – 1.34 (m, 1H), 0.96 (d, J = 6.6 Hz, 3H), 0.94 (d, J = 6.6 Hz, 3H), 0.44 (s, 3H).

^{13}C NMR (101 MHz, $CDCl_3$) δ 157.9, 143.4, 131.5, 130.6, 124.4, 113.3, 79.4, 71.6, 56.3, 55.3, 51.9, 46.8, 39.3, 28.7, 23.4, 23.0, 22.1, 14.1.

HRMS m/z (APCI): calc. for $C_{20}H_{27}O^+$ $[M+H-H_2O]^+$: 283.2056, found: 283.2050.

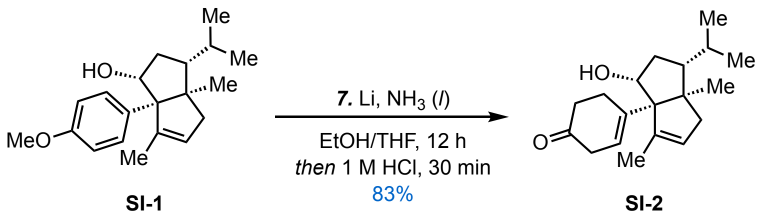

To a 250 mL three-necked round bottom flask with a Dewar condenser (cooled to −78 °C) was charged with condensed ammonia gas (~80 mL). Lithium metal (870 mg, 125.42 mmol, 15.0 equiv.) was carefully added to the stirred solution of ammonia. The resulting dark blue solution was stirred for 10 min at −78 °C before a solution of **SI-1** (2.51 g, 8.36 mmol, 1.0 equiv) in THF (42 mL) was added. The reaction mixture was stirred for 30 min at −78 °C before EtOH (1.48 mL, 25.08 mmol, 2.0 equiv.) was added at this temperature. The reaction mixture was slowly warmed to −50 °C and then stirred overnight at this temperature before it was carefully quenched with MeOH (20 mL) and saturated aqueous NH_4Cl (20 mL). The resulting suspension was slowly warmed to room temperature under a gentle stream of argon gas to aid in the removal of ammonia. After removal of ammonia, an aqueous solution of 1.0 M HCl (80 mL) was added at 0 °C and stirred for 30 min. The mixture was then poured into an Erlenmeyer flask containing saturated aqueous $NaHCO_3$ (150 mL) to quench. The resulting mixture was extracted with EtOAc (3 × 80 mL). The combined organic phase was washed with brine (100 mL), dried over anhydrous Na_2SO_4, filtered and concentrated under reduced pressure. The crude residue was purified by flash chromatography (10 to 30% ethyl acetate in hexanes) to afford **SI-2** (2.0 g, 83%) as a colorless oil.

R_f = 0.4 (hexane/ethyl acetate = 3/1).
$[\alpha]_D^{24}$ = +0.5 (*c* = 0.2 in MeOH).
IR (film) λ_{max}: 3457, 3032, 2956, 2870, 2844, 1708, 1456, 1401, 1090, 820, 724 cm^{-1}.
^{1}H NMR (400 MHz, $CDCl_3$) δ 5.46 – 5.42 (m, 1H), 5.22 (dt, *J* = 3.0, 1.5 Hz, 1H), 4.02 (dd, *J* = 11.4, 6.2 Hz, 1H), 2.94 – 2.89 (m, 2H), 2.69 – 2.55 (m, 1H), 2.45 – 2.26 (m, 4H), 2.07 (ddd, *J* = 11.2, 6.2, 4.8 Hz, 1H), 2.01 – 1.89 (m, 2H), 1.71 – 1.59 (m, 4H), 1.57 – 1.47 (m, 1H), 1.33 – 1.24 (m, 1H), 0.97 (s, 3H), 0.94 (d, *J* = 6.6 Hz, 3H), 0.90 (d, *J* = 6.6 Hz, 3H).
^{13}C NMR (101 MHz, $CDCl_3$) δ 212.5, 143.5, 139.8, 124.2, 122.4, 78.0, 72.8, 56.7, 51.9, 46.8, 40.4, 39.0, 38.7, 28.7, 28.6, 23.4, 23.0, 20. 9, 14.0.
HRMS m/z (APCI): calc. for $C_{19}H_{29}O_2^+$ $[M+H]^+$: 289.2162, found: 289.2158.

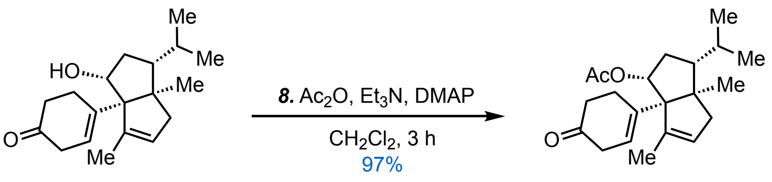

SI-2 **26**

To a mixture of **SI-2** (1.86 g, 6.45 mmol, 1.0 equiv.) and DMAP (788 mg, 0.65 mmol, 0.1 equiv.) in DCM (65 mL) were added Ac_2O (0.73 mL, 7.74 mmol, 1.2 equiv.) and Et_3N (1.1 mL, 8.39 mmol, 1.3 equiv.) at 0 ˚C. The reaction mixture was warmed to room temperature and stirred for 3 h. Upon full consumption of the starting material as indicated by TLC, the reaction was quenched with saturated aqueous $NaHCO_3$ (80 mL). The resulting mixture was extracted with DCM (3 × 60 mL). The combined organic phase was washed with brine, dried over Na_2SO_4, and concentrated under reduced pressure. The crude residue was purified by flash chromatography (10 to 20% ethyl acetate in hexanes) to afford **26** (2.07 g, 97%) as a colorless oil.

R$_f$ = 0.4 (hexane/ethyl acetate = 5/1).

$[\alpha]_D^{24}$ = +14.0 (c = 0.2 in MeOH).

IR (film) λ_{max}: 2968, 2872, 1734, 1722, 1366, 1260, 1236, 1154, 793, 760 cm^{-1}.

^{1}H NMR (400 MHz, $CDCl_3$) δ 5.37 – 5.32 (m, 1H), 5.26 – 5.21 (m, 1H), 4.97 (dd, J = 11.0, 6.4 Hz, 1H), 2.93 – 2.89 (m, 2H), 2.70 – 2.58 (m, 1H), 2.47 – 2.21 (m, 5H), 2.01 (s, 3H), 2.01 – 1.90 (m, 1H), 1.69 – 1.62 (m, 1H), 1.58 – 1.52 (m, 3H), 1.52 – 1.40 (m, 1H), 1.40 – 1.32 (m, 1H), 1.01 (s, 3H), 0.95 (d, J = 6.6 Hz, 3H), 0.89 (d, J = 6.6 Hz, 3H).

^{13}C NMR (101 MHz, $CDCl_3$) δ 212.3, 170.3, 143.0, 139.4, 124.6, 122.3, 80.1, 71.9, 56.7, 51.7, 46.3, 40.5, 38.8, 36.2, 28.9, 28.5, 23.4, 22.8, 21.6, 20.4, 13.7.

HRMS m/z (ESI): calc. for $C_{21}H_{30}O_3{}^+Na$ $[M+Na]^+$: 353.2087, found: 353.2082.

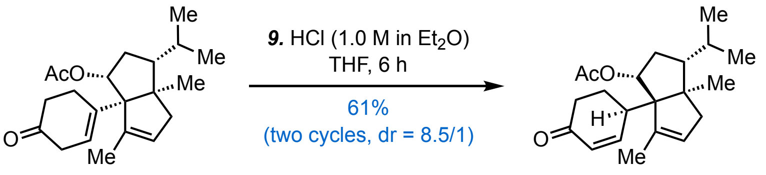

26 **27**

To a solution of **26** (1.79 g, 5.42 mmol, 1.0 equiv.) in THF (54 mL) was added a solution of HCl (1.0 M in Et_2O, 27.1 mL, 27.1 mmol, 5.0 equiv.) at 0 ˚C. The reaction mixture was stirred for 6 h at 0 ˚C before it was

carefully poured into an Erlenmeyer flask containing saturated aqueous $NaHCO_3$ (60 mL) to quench. The resulting mixture was extracted with EtOAc (3 × 60 mL). The combined organic phase was washed with brine (100 mL), dried over anhydrous Na_2SO_4, filtered and concentrated under reduced pressure. The crude residue was purified by flash chromatography (10 to 20% ethyl acetate in hexanes) to afford a mixture of diastereomers of **27** (570 mg, 32%, 8.8/1 dr) as a colorless oil and recovered starting material (1.22 g, 68%). The recovered SM (1.22 g, 3.7 mmol) was processed again following the same steps, yielding more of the desired product (350 mg, 29%, 8/1 dr) and recovered **26** (770 mg, contaminated with some impurities). After two cycles, **27** (920 mg, 61%, 8.5/1 dr) was obtained.

Note: When the SM is less than 770 mg for the third cycle, the reaction time must be shortened, otherwise the dr value will be greatly reduced.

R*f* = 0.42 (hexane/ethyl acetate = 5/1).

$[\alpha]_D^{24}$ = +76.0 (*c* = 0.2 in MeOH).

IR (film) λ_{max}: 2959, 2953, 1742, 1682, 1382, 1374, 1364, 1237, 894. 670 cm^{-1}.

^{1}H NMR (400 MHz, $CDCl_3$) δ 7.30 – 7.17 (m, 1H, overlap with $CDCl_3$), 5.94 (ddd, *J* = 10.4, 3.1, 1.2 Hz, 1H), 5.30 – 5.24 (m, 1H), 5.08 (dd, *J* = 9.2, 7.0 Hz, 1H), 2.79 – 2.69 (m, 1H), 2.55 – 2.45 (m, 1H), 2.38 – 2.22 (m, 3H), 2.04 (s, 3H), 2.02 – 1.94 (m, 1H), 1.91 – 1.79 (m, 2H), 1.71 – 1.56 (m, 4H, overlap), 1.48 – 1.38 (m, 1H), 1.35 – 1.27 (m, 1H), 1.07 (s, 3H), 0.93 (d, *J* = 6.6 Hz, 3H), 0.86 (d, *J* = 6.6 Hz, 3H).

^{13}C NMR (101 MHz, $CDCl_3$) δ 199.7, 170.3, 156.8, 142.6, 128.1, 125.3, 78.1, 66.5, 55.3, 51.3, 46.2, 39.1, 39.0, 36.4, 28.4, 26.3, 23.8, 22.4, 21.5, 19.7, 13.4.

HRMS m/z (ESI): calc. for $C_{21}H_{30}O_3{}^+Na$ $[M+Na]^+$: 353.2087, found: 353.2085.

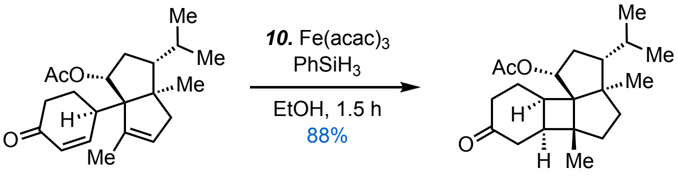

27 **28**

To a mixture of **27** (900 mg, 2.73 mmol, 1.0 equiv.) and $Fe(acac)_3$ (963 mg, 2.73 mmol, 1.0 equiv.) in EtOH (55 mL) was added $PhSiH_3$ (841 μL, 6.82 mmol, 2.5 equiv.) at 60 ˚C. The reaction mixture was stirred for 1.5 h at 60 ˚C. Upon full consumption of starting material as indicated by TLC, the reaction mixture was filtered through a short silica gel column and washed with EtOAc. The combined filtrate was concentrated

under reduced pressure. The crude residue was purified by flash chromatography (10 to 20% ethyl acetate in hexanes) to afford product **28** (798 mg, 88%) as a white foam.

Note: $PhSiH_3$ should be introduced after the mixture has reached and stabilized at 60 ˚C.

$\mathbf{R}_f$ = 0.41 (hexane/ethyl acetate = 5/1).

[α]$_D^{24}$ = +43.0 (*c* = 0.2 in MeOH).

IR (film) λ_{max}: 2954, 1732, 1716, 1462, 1376, 1245, 1167, 1130, 949 cm^{-1}.

^{1}H NMR (400 MHz, $CDCl_3$) δ 5.19 (dd, *J* = 7.1, 1.8 Hz, 1H), 2.54 (dd, *J* = 14.7, 12.6 Hz, 1H), 2.43 – 2.31 (m, 2H), 2.30 – 2.14 (m, 2H), 2.00 (s, 3H), 2.05 – 1.60 (m, 7H), 1.53 – 1.45 (m, 1H), 1.38 – 1.18 (m, 3H), 1.06 (s, 3H), 1.03 (s, 3H), 0.98 (d, *J* = 6.4 Hz, 3H), 0.84 (d, *J* = 6.4 Hz, 3H).

^{13}C NMR (101 MHz, $CDCl_3$) δ 213.7, 170.8, 77.5, 64.6, 55.8, 49.2, 47.1, 40.5, 40.4, 38.9, 38.0, 36.3, 35.6, 31.5, 30.9, 23.4, 23.1, 23.0, 21.9, 19.4, 15.4.

HRMS m/z (ESI): calc. for $C_{21}H_{32}O_3{}^+Na$ $[M+Na]^+$: 355.2244, found: 355.2240.

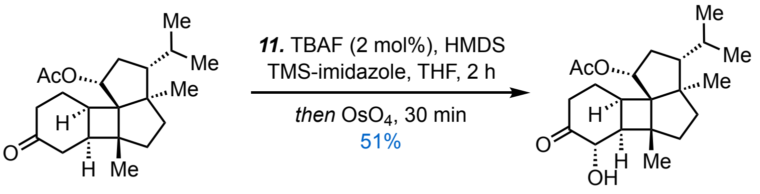

28 **29**

To a solution of **28** (770 mg, 2.32 mmol, 1.0 equiv.) in THF (23 mL) were added HMDS (1.5 mL, 6.95 mmol, 3.0 equiv.) and TMS-imidazole (1.02 mL, 6.95 mmol, 3.0 equiv.) at room temperature. Then a solution of TBAF (1.0 M in THF, 46 μL, 0.046 mmol, 0.02 equiv.) was added and the mixture was stirred for 2 h at room temperature. Upon full consumption of starting material as indicated by TLC, an aqueous solution of OsO_4[2] (0.16 M in H_2O, 17.4 mL, 2.78 mmol, 1.2 equiv.) was added dropwise at room temperature. After 30 min, the reaction was quenched by adding $NaHSO_3$ (20 mL). The resulting mixture was stirred for 30 min and then extracted with EtOAc (3 × 50 mL). The combined organic phase was washed with brine (80 mL), dried over anhydrous Na_2SO_4, filtered and concentrated under reduced pressure. The crude residue was purified by flash chromatography (13 to 30% ethyl acetate in hexanes) to afford **29** (412 mg, 51%) as a white foam, and **28** (123 mg, 16%) was recovered.

Note: OsO_4 is a highly toxic chemical and should be used with caution in a well-ventilated fume hood. Addition of catalytic amount TBAF significantly accelerates the silyl enol ether formation reaction.

R_f = 0.35 (hexane/ethyl acetate = 3/1).

[α]$_D^{24}$ = +75.7 (*c* = 0.2 in MeOH).

IR (film) λ_{max}: 3481, 2954, 2870, 1717, 1463, 1376, 1246, 1126, 1108, 735, 726 cm^{-1}.

^{1}H NMR (400 MHz, CDCl$_3$) δ 5.26 (dd, *J* = 7.1, 1.7 Hz, 1H), 4.54 (d, *J* = 10.9 Hz, 1H), 3.35 (s, 1H), 2.56 (dt, *J* = 19.7, 3.3 Hz, 1H), 2.44 – 2.16 (m, 3H), 2.00 (s, 3H), 1.93 – 1.84 (m, 3H), 1.81 – 1.53 (m, 5H), 1.41 – 1.25 (m, 2H), 1.25 (s, 3H), 1.05 (s, 3H), 0.98 (d, *J* = 6.4 Hz, 3H), 0.85 (d, *J* = 6.5 Hz, 3H).

^{13}C NMR (101 MHz, CDCl$_3$) δ 213.1, 170.7, 77.2, 73.4, 64.5, 55.6, 49.2, 47.7, 43.6, 40.6, 40.3, 36.9, 36.3, 31.5, 31.2, 23.1, 23.0, 22.2, 21.9, 19.5, 15.4.

HRMS m/z (ESI): calc. for $C_{21}H_{32}O_4{}^+Na$ [M+Na]$^+$: 371.2193, found: 371.2184.

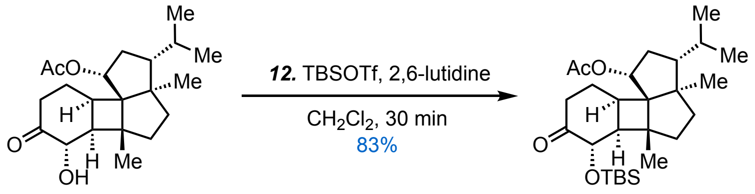

29 **SI-3**

To a solution of **29** (370 mg, 1.06 mmol, 1.0 equiv.) in DCM (11 mL) were added 2,6-lutidine (742 μL, 6.36 mmol, 6.0 equiv.) and TBSOTf (910 μL, 5.24 mmol, 4.0 equiv.) at −78 °C. The reaction was stirred for 30 min at −78 °C. Upon full consumption of starting material as indicated by TLC, the reaction was quenched with saturated aqueous $NaHCO_3$ (10 mL) and extracted with ethyl acetate (3 × 20 mL). The combined organic phase was dried over anhydrous Na_2SO_4, filtered and concentrated under reduced pressure. The crude residue was purified by flash-column chromatography on silica gel (5 to 10% ethyl acetate in hexane) to afford **SI-3** (407 mg, 83%) as a yellow oil.

R_f = 0.4 (hexane/ethyl acetate = 8/1).

[α]$_D^{24}$ = +44.0 (*c* = 0.2 in MeOH).

IR (film) λ_{max}: 2954, 2930, 1731, 1471, 1375, 1248, 1151, 1137, 836, 778 cm^{-1}.

^{1}H NMR (400 MHz, CDCl$_3$) δ 5.22 (dd, *J* = 7.1, 1.8 Hz, 1H), 4.63 (d, *J* = 10.5 Hz, 1H), 2.51 – 2.23 (m, 3H), 2.23 – 2.09 (m, 1H), 1.99 (s, 3H), 1.93 – 1.80 (m, 3H), 1.75 – 1.46 (m, 5H), 1.40 – 1.22 (m, 2H), 1.21 (s, 3H), 1.05 (s, 3H), 0.97 (d, *J* = 6.5 Hz, 3H), 0.87 (s, 9H), 0.84 (d, *J* = 6.5 Hz, 3H), 0.14 (s, 3H), 0.01 (s, 3H).

^{13}C NMR (101 MHz, CDCl$_3$) δ 211.5, 170.8, 77.4, 75.2, 63.9, 55.3, 49.5, 47.9, 43.9, 40.5, 40.1, 38.3, 36.4, 32.0, 31.4, 26.1, 23.1, 23.1, 23.0, 21.9, 19.6, 18.5, 15.6, −3.1, −4.8.
HRMS m/z (APCI): calc. for $C_{27}H_{45}O_4Si^-$ [M–H]$^-$: 461.3093, found: 461.3096.

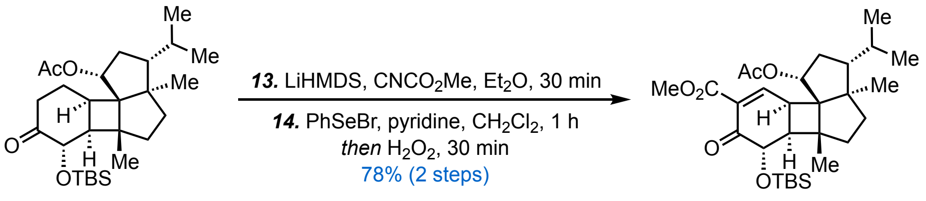

SI-3 **30**

To a solution of **SI-3** (355 mg, 0.77 mmol, 1.0 equiv.) in Et_2O (15 mL) was added a solution of LiHMDS (1.0 M in THF, 1.9 mL, 1.9 mmol, 2.5 equiv.) at −78 °C. The reaction mixture was stirred for 30 min at −78 °C, then warmed to −40 °C and stirred for an additional 30 min. The reaction was cooled back to −78 °C, and Mander's reagent (151 µL, 1.9 mmol, 2.5 equiv.) was added, followed by HMPA (319 µL, 1.9 mmol, 2.5 equiv.). The resulting mixture was stirred for 30 min at −78 °C, then warmed to −40 °C and stirred for an additional 30 min at this temperature. Upon full consumption of starting material as indicated by TLC, the reaction mixture was quenched with saturated aqueous $NaHCO_3$ (20 mL) and extracted with EtOAc (3 × 30 mL). The combined organic phase was washed with brine (30 mL), dried over anhydrous Na_2SO_4, filtered and concentrated under reduced pressure. The resulting residue was filtered through a short silica gel column and washed with 15% ethyl acetate in hexanes. The combined filtrate was concentrated under reduced pressure to afford crude product which was used without further purification in the next step.

To a mixture of the above crude product in DCM (8 mL) was added pyridine (186 µL, 2.31 mmol, 3.0 equiv.) at room temperature, followed by a dropwise addition of a solution of PhSeBr (363 mg, 1.54 mmol, 2.0 equiv.) in DCM (4 mL). The reaction mixture was stirred for 1 h at room temperature. Upon full consumption of starting material as indicated by TLC, a solution of H_2O_2 (30 wt.% in water, 262 µL, 2.31 mmol, 3.0 equiv.) was added dropwise. The reaction was stirred for an additional 30 min before it was quenched with $Na_2S_2O_3$ (10 mL) and extracted with DCM (3 × 10 mL). The combined organic phase was washed with brine (20 mL), dried over anhydrous Na_2SO_4, filtered and concentrated under reduced pressure. The crude residue was purified by flash-column chromatography on silica gel (5 to 12% ethyl acetate in hexane) to afford **30** (311 mg, 78%) as a white foam.
Note: H_2O_2 should be handled with extra caution especially on large scale due to its explosive nature.

R_f = 0.35 (hexane/ethyl acetate = 8/1).

$[\alpha]_D^{24}$ = −35.1 (c = 0.2 in MeOH).

IR (film) λ_{max}: 2953, 2929, 1736, 1472, 1435, 1376, 1274, 1130, 858, 778 cm^{-1}.

^{1}H NMR (400 MHz, $CDCl_3$) δ 7.02 (dd, J = 4.1, 2.4 Hz, 1H), 5.02 (dd, J = 7.5, 2.5 Hz, 1H), 4.41 (d, J = 8.0 Hz, 1H), 3.75 (s, 3H), 3.08 (dd, J = 8.9, 4.8 Hz, 1H), 2.41 (ddd, J = 15.1, 9.5, 7.6 Hz, 1H), 2.24 – 2.15 (m, 1H), 2.02 (s, 3H), 2.07 – 1.95 (m, 1H, overlap), 1.84 – 1.57 (m, 4H), 1.40 – 1.31 (m, 1H), 1.23 – 1.13 (m, 1H), 1.10 (s, 3H), 1.07 (s, 3H), 0.97 (d, J = 6.5 Hz, 3H), 0.86 (s, 9H), 0.84 (d, J = 6.5 Hz, 3H), 0.12 (s, 3H), 0.07 (s, 3H).

^{13}C NMR (101 MHz, $CDCl_3$) δ 195.3, 170.5, 164.4, 156.5, 128.4, 75.8, 74.8, 70.0, 56.2, 52.2, 48.9, 47.0, 46.3, 40.4, 40.2, 36.3, 33.2, 31.5, 26.0, 23.0, 22.7, 21.5, 20.1, 18.5, 15.6, −3.5, −4.4.

HRMS m/z (APCI): calc. for $C_{29}H_{45}O_6Si^-$ $[M–H]^-$: 517.2991, found: 517.2998.

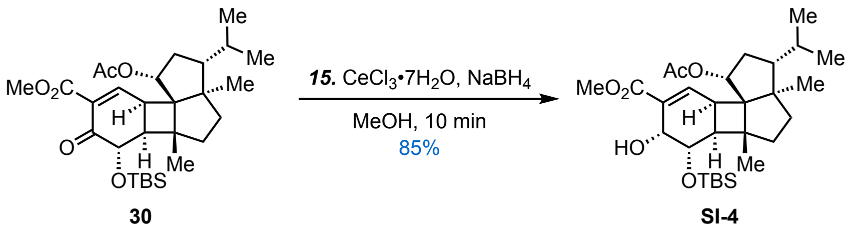

To a mixture of **30** (280 mg, 0.54 mmol, 1.0 equiv.) and $CeCl_3·7H_2O$ (1.01 g, 2.7 mmol, 5.0 equiv.) in MeOH (10 mL) was added $NaBH_4$ (42 mg, 1.08 mmol, 2.0 equiv.) at 0 °C. The reaction was stirred for 30 min before it was carefully quenched with saturated aqueous $NaHCO_3$ (20 mL) and extracted with EtOAc (3 × 30 mL). The combined organic phase was dried over anhydrous Na_2SO_4, filtered and concentrated under reduced pressure. The crude residue was purified by flash-column chromatography on silica gel (10 to 12% ethyl acetate in hexane) to afford **SI-4** (239 mg, 85%) as a white foam.

R_f = 0.34 (hexane/ethyl acetate = 8/1).

$[\alpha]_D^{24}$ = −56.5 (c = 0.2 in MeOH).

IR (film) λ_{max}: 3525, 2954, 2935, 1737, 1695, 1376, 1278, 1243, 1083, 881, 777 cm^{-1}.

^{1}H NMR (400 MHz, $CDCl_3$) δ 7.02 (dd, J = 4.5, 1.5 Hz, 1H), 5.07 (dd, J = 7.6, 2.4 Hz, 1H), 4.20 (dt, J = 6.7, 1.7 Hz, 1H), 3.92 (dd, J = 8.2, 6.7 Hz, 1H), 3.73 (s, 3H), 3.69 (s, 1H br.), 2.86 (ddd, J = 9.8, 4.5, 2.2 Hz, 1H), 2.40 (ddd, J = 15.0, 9.4, 7.5 Hz, 1H), 2.01 (s, 3H), 2.00 – 1.88 (m, 2H), 1.83 – 1.53 (m, 4H), 1.36

– 1.28 (m, 1H), 1.15 (s, 3H), 1.19 – 1.10 (m, 1H, overlap with Me group), 1.02 (s, 3H), 0.97 (d, J = 6.5 Hz, 3H), 0.85 (s, 9H), 0.84 (d, J = 6.5 Hz, 3H), 0.13 (s, 3H), 0.11 (s, 3H).

^{13}C NMR (101 MHz, $CDCl_3$) δ 170.7, 167.3, 142.7, 129.5, 76.2, 73.9, 69.0, 69.0, 56.1, 51.8, 48.9, 47.2, 44.9, 41.1, 40.5, 36.2, 33.6, 31.6, 26.4, 23.1, 22.7, 21.5, 21.1, 18.6, 15.4, −2.9, −4.5.

HRMS m/z (APCI): calc. for $C_{29}H_{47}O_6Si^-$ $[M–H]^-$: 519.3147, found: 519.3147.

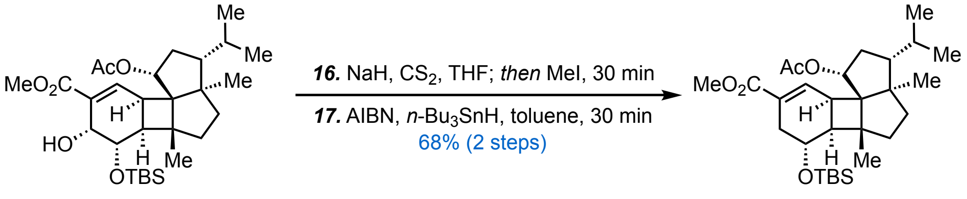

SI-4 **31**

To a mixture of **SI-4** (186 mg, 0.36 mmol, 1.0 equiv.) and CS_2 (130 µL, 2.14 mmol, 6.0 equiv.) in THF (3.6 mL) was added NaH (60% dispersion in mineral oil, 72 mg, 1.8 mmol, 5.0 equiv.) at room temperature. The reaction mixture was warmed to 60 °C and stirred for 30 min at this temperature, then it was cooled down to room temperature and MeI (134 µL, 2.14 mmol, 6.0 equiv.) was added. The reaction was stirred for 10 min at room temperature before it was carefully quenched with $NaHCO_3$ (5 mL) and extracted with EtOAc (3 × 100 mL). The combined organic phase was washed with brine (10 mL), dried over anhydrous Na_2SO_4, filtered and concentrated under reduced pressure. The resulting residue was filtered through a short silica gel column and washed with 10% ethyl acetate in hexanes. The combined filtrate was concentrated under reduced pressure to afford crude product which was used without further purification in the next step.

To a mixture of the above crude product in toluene (4 mL) were added AIBN (30 mg, 0.18 mmol, 0.5 equiv.) and n-Bu_3SnH (288 µL, 1.07 mmol, 3.0 equiv.) at room temperature. The reaction mixture was degassed three times before it was moved to an oil bath preheated to 80 °C and stirred for 30 min at this temperature. Upon full consumption of starting material as indicated by TLC, the crude residue was purified directly by flash-column chromatography on silica gel (5 to 10% ethyl acetate in hexane) to afford **31** (123 mg, 68%) as a white foam.

R_f = 0.5 (hexane/ethyl acetate = 8/1).

$[\alpha]_D^{24}$ = −30.6 (c = 0.2 in MeOH).

IR (film) λ_{max}: 2954, 2929, 1737, 1716, 1376, 1251, 1170, 1107, 851, 776 cm^{-1}.

^{1}H NMR (400 MHz, CDCl$_3$) δ 7.02 (dd, J = 4.1, 2.4 Hz, 1H), 5.09 (dd, J = 7.6, 2.4 Hz, 1H), 3.95 (td, J = 9.0, 4.4 Hz, 1H), 3.70 (s, 3H), 2.92 (dt, J = 9.5, 3.5 Hz, 1H), 2.66 (dd, J = 15.7, 4.5 Hz, 1H), 2.38 (ddd, J = 15.0, 9.5, 7.5 Hz, 1H), 2.01 (s, 3H), 1.99 – 1.84 (m, 3H), 1.80 – 1.58 (m, 4H), 1.38 – 1.26 (m, 1H), 1.18 – 1.10 (m, 1H), 1.09 (s, 3H), 1.03 (s, 3H), 0.97 (d, J = 6.5 Hz, 3H), 0.86 (s, 9H), 0.84 (d, J = 6.5 Hz, 3H), 0.11 (s, 3H), 0.08 (s, 3H).
^{13}C NMR (101 MHz, CDCl$_3$) δ 170.8, 167.2, 141.4, 126.7, 76.1, 69.8, 69.4, 56.2, 51.6, 49.0, 47.5, 47.2, 40.9, 40.4, 36.5, 34.9, 31.6, 31.6, 26.1, 23.1, 22.7, 21.5, 21.1, 18.1, 15.5, −3.0, −4.1.
HRMS m/z (APCI): calc. for $C_{29}H_{47}O_5Si^-$ $[M–H]^-$: 503.3179, found: 503.3171.

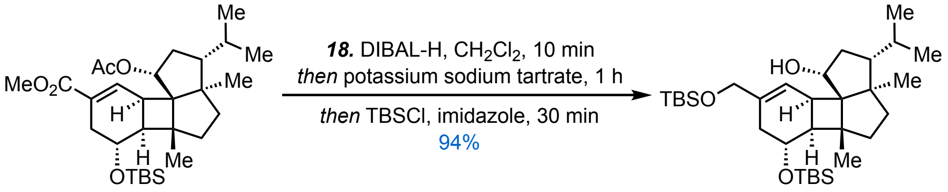

31 **32**

To a solution of **31** (113 mg, 0.23 mmol, 1.0 equiv.) in DCM (3 mL) was added DIBAL-H (1.0 M in toluene, 1.15 mL, 1.15 mmol, 5.0 equiv.) at −78 °C. The reaction was stirred for 10 min at −78 °C. Upon full consumption of starting material as indicated by TLC, potassium sodium tartrate (325 mg, 1.15 mmol, 5.0 equiv.) was added at −78 °C. Then the reaction mixture was warmed to 0 °C and stirred for 1h at this temperature. To the resulting mixture was added imizazole (157 mg, 2.3 mmol, 10.0 equiv.) and TBSCl (173 mg, 1.15 mmol, 5.0 equiv.) at 0 °C. The reaction mixture was stirred for 1 h at 0 °C before it was quenched with saturated aqueous $NaHCO_3$ (5 mL) and extracted with DCM (3 × 10 mL). The combined organic phase was dried over anhydrous Na_2SO_4, filtered and concentrated under reduced pressure. The crude residue was purified by flash-column chromatography on silica gel (5 to 10% ethyl acetate in hexane) to afford **32** (119 mg, 94%) as a pale-yellow oil.
Note: potassium sodium tartrate must be added and stirred for 1 h at 0 °C prior to the addition of imidazole and TBSCl.

R_f = 0.5 (hexane/ethyl acetate = 10/1).
$[\alpha]_D^{24}$ = −22.5 (c = 0.2 in MeOH).
IR (film) λ_{max}: 3575, 2952, 2927, 2857, 1472, 1462, 1253, 1083, 1073, 833, 773 cm^{-1}.

^{1}H NMR (400 MHz, CDCl$_3$) δ 5.85 – 5.72 (m, 1H), 4.24 (dd, J = 7.6, 2.3 Hz, 1H), 4.07 (td, J = 8.8, 4.5 Hz, 1H), 4.00 (q, J = 2.1 Hz, 2H), 2.87 – 2.79 (m, 1H), 2.31 – 2.18 (m, 2H), 1.95 – 1.81 (m, 3H), 1.76 – 1.57 (m, 5H), 1.39 (ddd, J = 14.6, 10.3, 2.4 Hz, 1H), 1.13 – 1.03 (m, 1H), 1.03 (s, 3H), 1.02 (s, 3H), 0.97 (d, J = 6.5 Hz, 3H), 0.90 (s, 9H), 0.87 (s, 9H), 0.86 (d, J = 6.5 Hz, 3H), 0.09 (s, 3H), 0.07 (s, 3H), 0.06 (s, 6H).

^{13}C NMR (101 MHz, CDCl$_3$) δ 138.2, 121.3, 74.8, 70.8, 70.2, 66.7, 55.8, 49.3, 47.5, 46.8, 41.1, 36.8, 34.3, 34.0, 31.7, 26.1, 26.1, 23.3, 22.9, 21.3, 18.5, 18.1, 16.1, −3.0, −4.1, −5.2, −5.2.

HRMS m/z (ESI): calc. for $C_{32}H_{60}O_3Si_2^+Na$ [M+Na]$^+$: 571.3973, found: 571.3969.

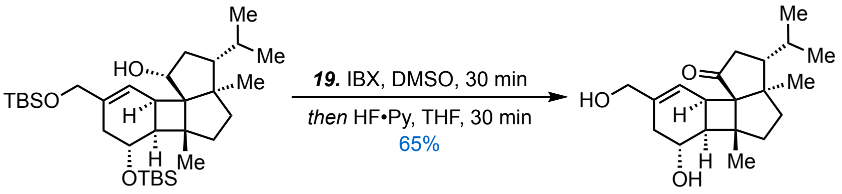

32

(−)-Psathyrin A (**1**)

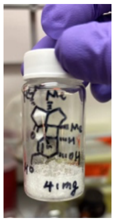

To a solution of **32** (109 mg, 0.2 mmol, 1.0 equiv.) in DMSO (2 mL) was added IBX (168 mg, 0.6 mmol, 3.0 equiv.) at room temperature. The reaction mixture was heated to 60 °C and stirred for 30 min. Upon full consumption of starting material as indicated by TLC, the reaction was allowed to cool to room temperature. HF·Py (approx.70% HF, 667 μL) was then added, and the mixture was stirred for 30 min. The reaction mixture was then carefully poured into an Erlenmeyer flask containing saturated aqueous $NaHCO_3$ (100 mL) to quench and extracted with EtOAc (3 × 40 mL). The combined organic phase was dried over anhydrous Na_2SO_4, filtered and concentrated under reduced pressure. The crude residue was purified by flash-column chromatography on silica gel (20 to 50% acetone in hexanes) to afford psathyrin A (41 mg, 65%) as a white solid.[3]

R_f= 0.3 (hexane/acetone = 1/1).

m.p.= 172–174 °C

[α]$_D^{24}$ = −56.0 (c = 0.27 in MeOH).

IR (film) λ_{max}: 3390, 2954, 2929, 2869, 1715, 1459, 1379, 1281, 1158, 1044, 735, 680 cm^{-1}.

^{1}H NMR (400 MHz, CDCl$_3$) δ 5.30 (br s, 1H), 4.06 – 3.98 (m, 1H), 4.00 – 3.87 (m, 2H), 2.91 (br d, J = 10.4, 1H), 2.34 – 2.16 (m, 3H), 2.05 – 1.78 (m, 6H), 1.75 – 1.64 (m, 1H), 1.60 – 1.50 (m, 1H), 1.13 (s, 3H), 1.08 (d, J = 6.5 Hz, 3H), 1.02 (s, 3H), 0.92 (d, J = 6.5 Hz, 3H).

^{13}C NMR (101 MHz, $CDCl_3$) δ 219.6, 137.2, 122.7, 74.5, 68.9, 66.9, 56.6, 54.8, 47.4, 46.7, 45.2, 42.2, 39.0, 34.4, 33.8, 32.2, 23.4, 23.0, 21.1, 14.8.

HRMS m/z (APCI): calc. for $C_{20}H_{29}O_3^-$ [M–H]$^-$: 317.2122, found: 317.2117.

Part 2. Natural Product NMR Data Comparison

Table S1. ^{1}H NMR (CD_3OD) Comparison of Natural & Our Synthetic Psathyrin A.

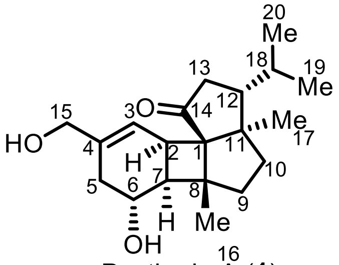

Psathyrin A (**1**)

No.	Natural, 600 MHz[3] δ (ppm, *J* in Hz)	Synthetic, 400 MHz δ (ppm, *J* in Hz)	Error (Syn. – Nat.)
2	2.91, br d (10.2)	2.91, br d (10.4)	0
3	5.30, br s	5.30, br s	0
5	2.24, m	2.34 – 2.16, m, overlapped	–
	1.83, m	2.05 – 1.78, m, overlapped	–
6	4.01, m	4.02, m	0.01
7	1.87, dd (10.2, 10.0)	2.05 – 1.78, m, overlapped	–
9	1.98, overlapped	2.05 – 1.78, m, overlapped	–
	1.90, m		
10	2.22, m	2.34 – 2.16, m, overlapped	–
	1.92 m	2.05 – 1.78, m, overlapped	–
12	1.55, m	1.55, m	0
13	2.27, dd (18.8, 8.1)	2.34 – 2.16, m	–
	1.98 (overlapped)	2.05 – 1.78, m	–
15	3.95, d (12.6)	3.96, d (12.9)	0.01
	3.91, d (12.6)	3.92, d (12.9)	0.01
16	1.13, s	1.13, s	0
17	1.01, s	1.02, s	0.01
18	1.69, m	1.69, m	0
19	1.08, d (6.5)	1.08, d (6.5)	0
20	0.91, d (6.5)	0.92, d (6.5)	0

Table S2. ^{13}C NMR (CD_3OD) Comparison of Natural & Our Synthetic Psathyrin A.

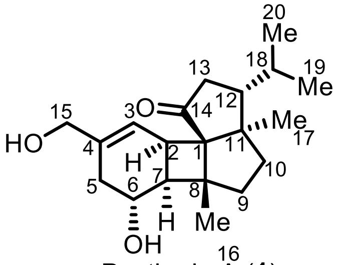

Psathyrin A (**1**)

No.	Natural, 151 MHz[3] δ (ppm)	Synthetic, 101 MHz δ (ppm)	Error (Syn. – Nat.)
1	74.5, C	74.5	0
2	33.7, CH	33.8	0.1
3	122.7, CH	122.7	0
4	137.2, C	137.2	0
5	34.3, CH_2	34.4	0.1
6	68.9, CH	68.9	0
7	47.4, CH	47.4	0
8	54.8, C	54.8	0
9	42.2, CH_2	42.2	0
10	39.0, CH_2	39.0	0
11	56.5, C	56.6	0.1
12	46.6, CH	46.7	0.1
13	45.2, CH_2	45.2	0
14	219.7. C	219.6	-0.1
15	67.9 (67.0[a]), CH_2	66.9	-0.1
16	21.1, CH_3	21.1	0
17	14.7, CH_3	14.8	0.1
18	32.2, CH_3	32.2	0
19	23.0, CH_3	23.0	0
20	23.4, CH_3	23.4	0

[a]The chemical shift for C15 in Table 1 of the isolation paper[3] is 67.9 ppm, which is inconsistent with the chemical shift of their spectrum, where 66.96 ppm is observed.

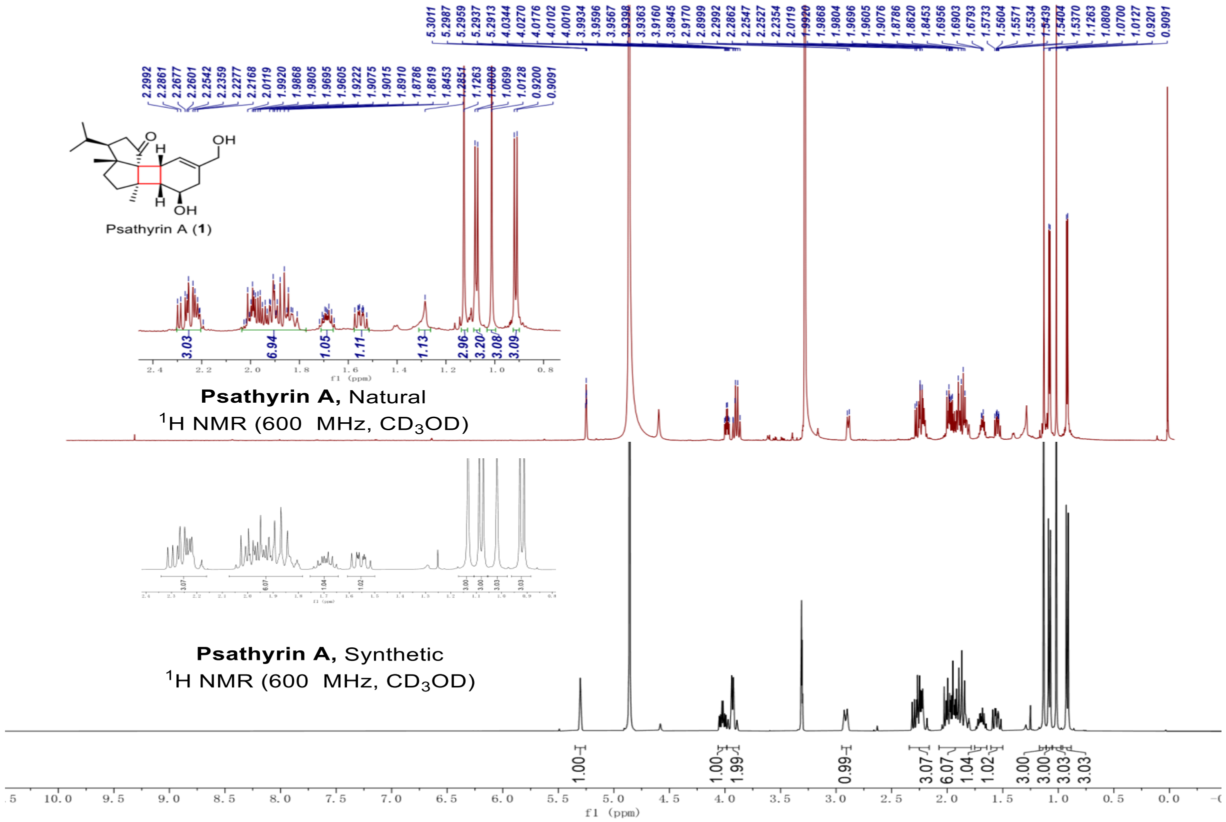
Psathyrin A (1)
Psathyrin A, Natural
1H NMR (600 MHz, CD3OD)
Psathyrin A, Synthetic
1H NMR (600 MHz, CD3OD)
f1 (ppm)

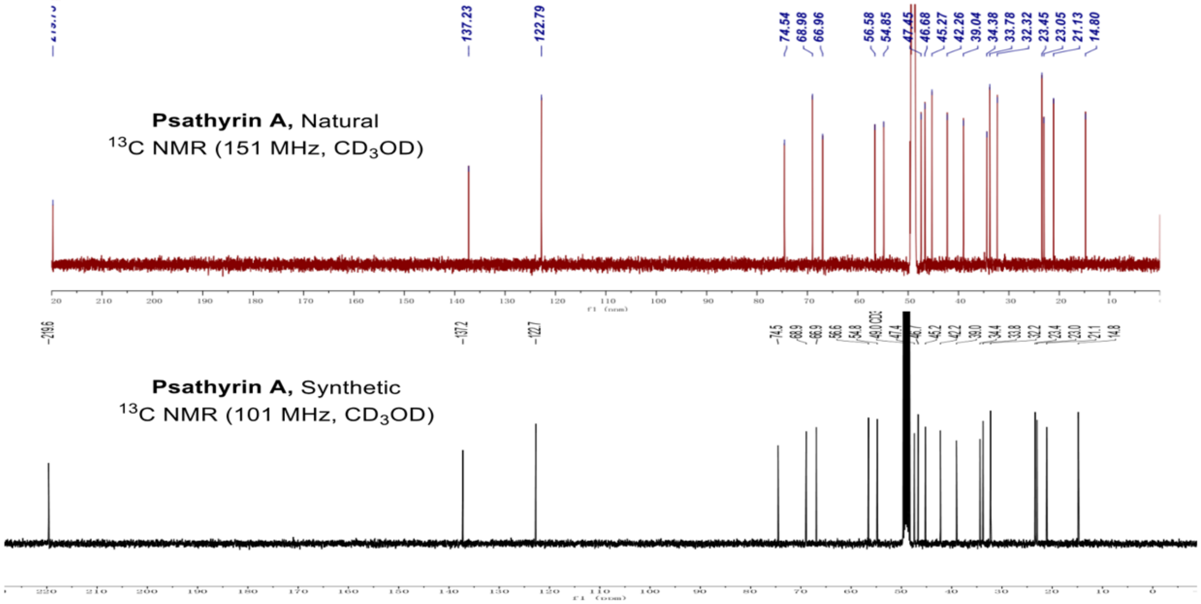
Psathyrin A, Natural
13C NMR (151 MHz, CD3OD)
Psathyrin A, Synthetic
13C NMR (101 MHz, CD3OD)
f1 (ppm)

Part 3. References

1. (a) Germain, N.; Alexakis, A. Formation of Contiguous Quaternary and Tertiary Stereocenters by Sequential Asymmetric Conjugate Addition of Grignard Reagents to 2-Substituted Enones and Mg−Enolate Trapping. *Chem. - Eur. J.* **2015**, *21*, 8597−8606. (b) Germain, N.; Guénée, L.; Mauduit, M.; Alexakis, A. Asymmetric Conjugate Addition to α-Substituted Enones/Enolate Trapping. *Org. Lett.* **2014**, *16*, 118−121. (c) Gerstner, N. C.; Schomaker, J. M. Stereocontrolled Synthesis of the Aminocyclopentitol Core of Jogyamycin via an Ichikawa Rearrangement Reaction. *J. Org. Chem.* **2019**, *84*, 14092–14100.

2. (a) McCormick, J. P.; Tomasik, W.; Johnson, M. W. α-Hydroxylation of Ketones: Osmium Tetroxide/N-Methylmorpholine-N-Oxide Oxidation of Silyl Enol Ethers. *Tetrahedron Lett.* **1981**, *22*, 607−610. (b) Zhao, W.; Zhang, D.; Wang, Y.; Yang, M. Total syntheses of rhodomollins A and B. *J. Am. Chem. Soc.* **2023**, *145*, 27160−27166.

3. Liu, Y.-P.; Dai, Q.; Wang, W.-X.; He, J.; Li, Z.-H.; Feng, T.; Liu, J.-K. Psathyrins: Antibacterial diterpenoids from *Psathyrella candolleana*. *J. Nat. Prod.* **2020**, *83*, 1725–1729.

Part 4. ^{1}H and ^{13}C NMR Spectra

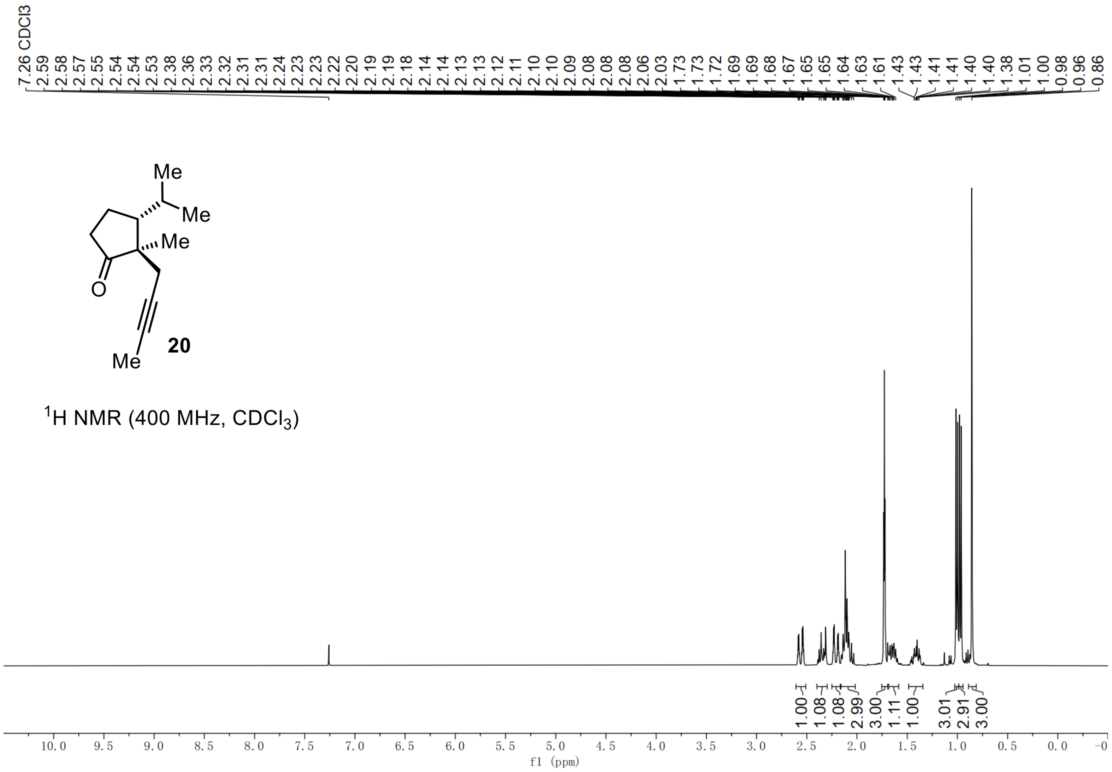

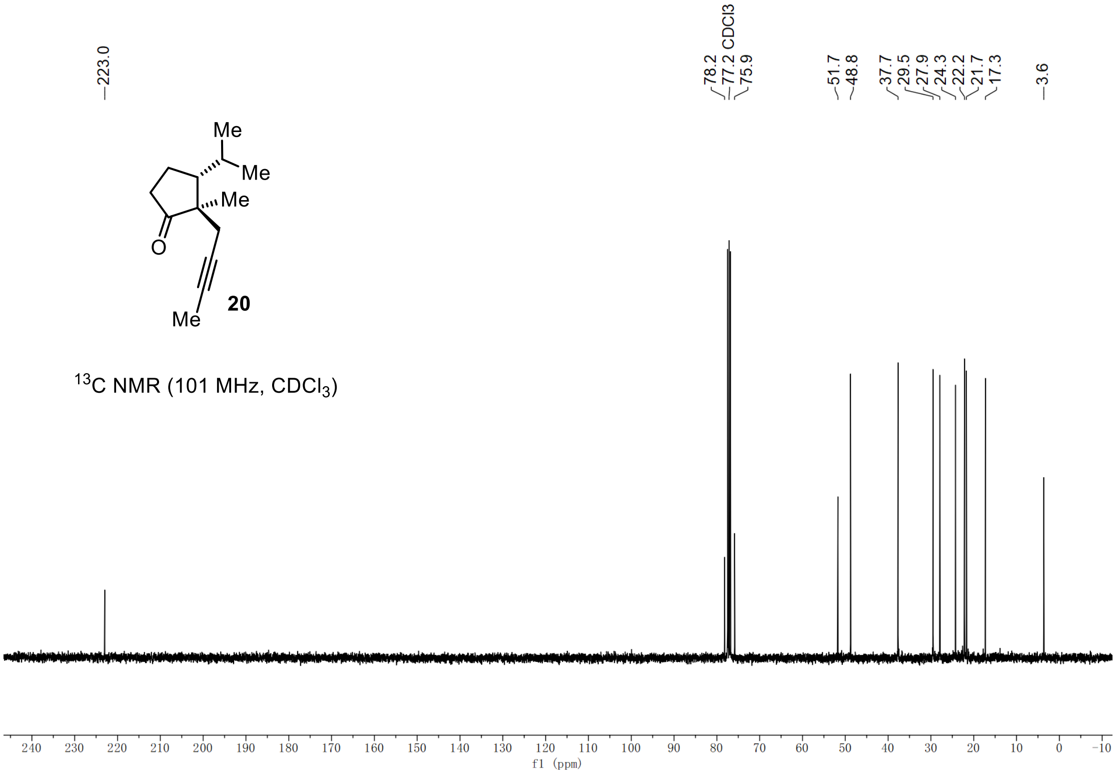

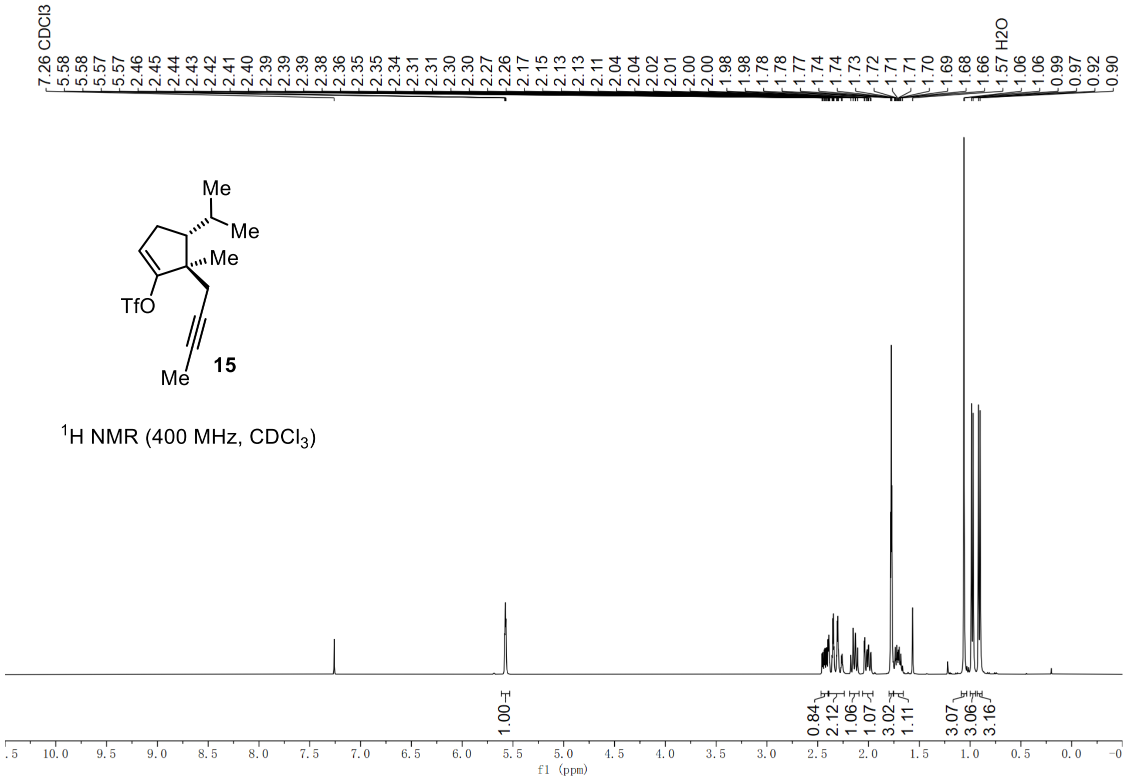
7.26 CDCl3
5.58
5.58
5.57
2.46
2.45
2.44
2.43
2.42
2.41
2.40
2.39
2.39
2.39
2.38
2.36
2.35
2.35
2.34
2.31
2.31
2.30
2.30
2.27
2.26
2.17
2.15
2.13
2.13
2.11
2.04
2.04
2.02
2.01
2.00
2.00
1.98
1.98
1.78
1.78
1.77
1.74
1.74
1.73
1.72
1.71
1.71
1.70
1.69
1.68
1.66
1.57 H2O
1.06
1.06
1.06
0.99
0.97
0.92
0.90
Me
Me
Me
TfO
Me
15
^{1}H NMR (400 MHz, CDCl$_3$)
1.00
0.84
2.12
1.06
1.07
3.02
1.11
3.07
3.06
3.16
f1 (ppm)

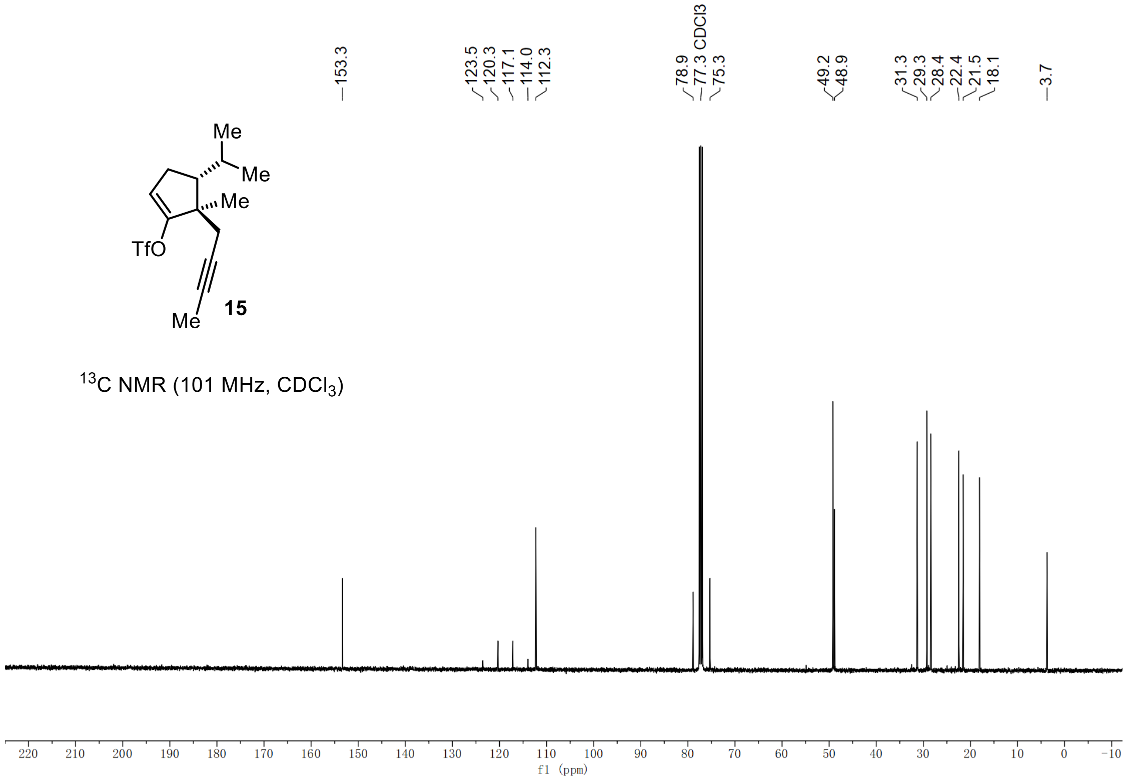
153.3
123.5
120.3
117.1
114.0
112.3
78.9
77.3 CDCl3
75.3
49.2
48.9
31.3
29.3
28.4
22.4
21.5
18.1
3.7
Me
Me
Me
TfO
Me
15
^{13}C NMR (101 MHz, CDCl$_3$)
f1 (ppm)

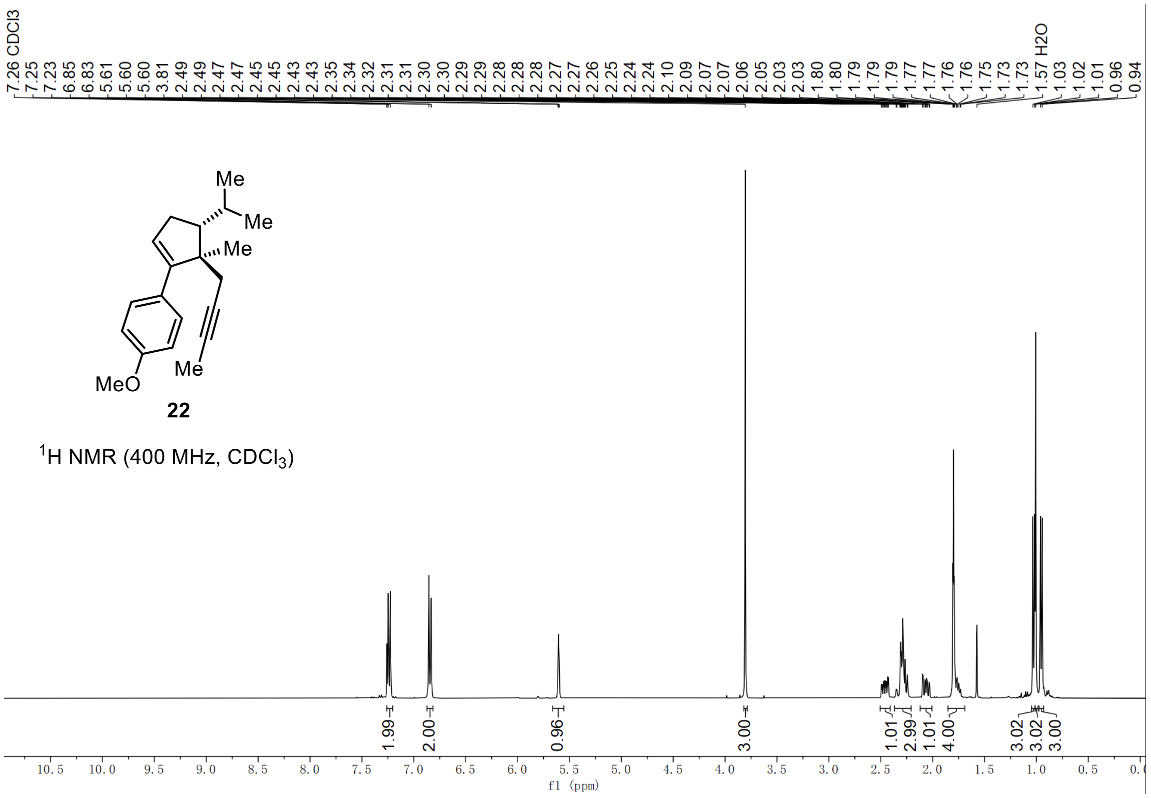
7.26 CDCl3
7.25
7.23
6.85
6.83
5.61
5.60
5.60
3.81
2.49
2.49
2.47
2.47
2.45
2.45
2.43
2.43
2.35
2.34
2.32
2.31
2.31
2.30
2.30
2.29
2.29
2.28
2.28
2.28
2.27
2.27
2.26
2.25
2.24
2.24
2.10
2.09
2.07
2.07
2.06
2.05
2.03
2.03
1.80
1.80
1.79
1.79
1.79
1.77
1.77
1.76
1.76
1.75
1.73
1.73
1.57 H2O
1.03
1.02
1.01
0.96
0.94
Me
Me
Me
Me
MeO
22
1H NMR (400 MHz, CDCl3)
1.99
2.00
0.96
3.00
1.01
2.99
1.01
4.00
3.02
3.02
3.00
f1 (ppm)

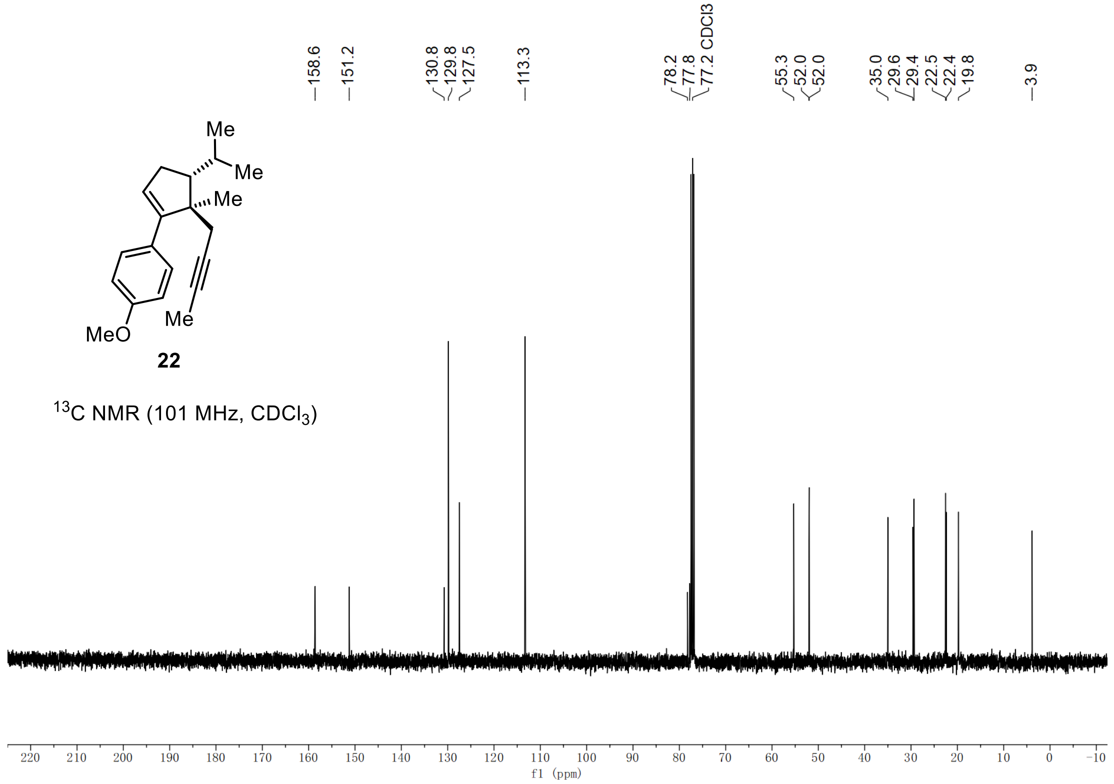
158.6
151.2
130.8
129.8
127.5
113.3
78.2
77.8
77.2 CDCl3
55.3
52.0
52.0
35.0
29.6
29.4
22.5
22.4
19.8
3.9
Me
Me
Me
Me
MeO
22
13C NMR (101 MHz, CDCl3)
f1 (ppm)

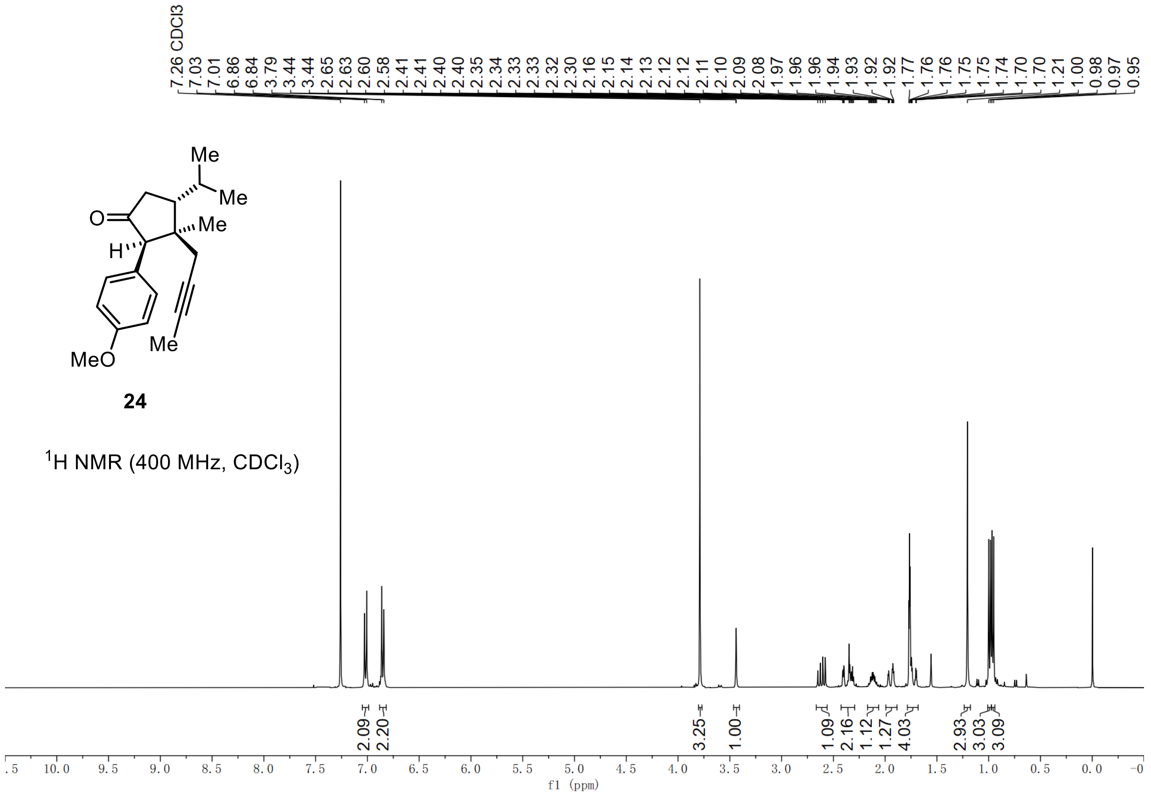

24

^{1}H NMR (400 MHz, $CDCl_3$)

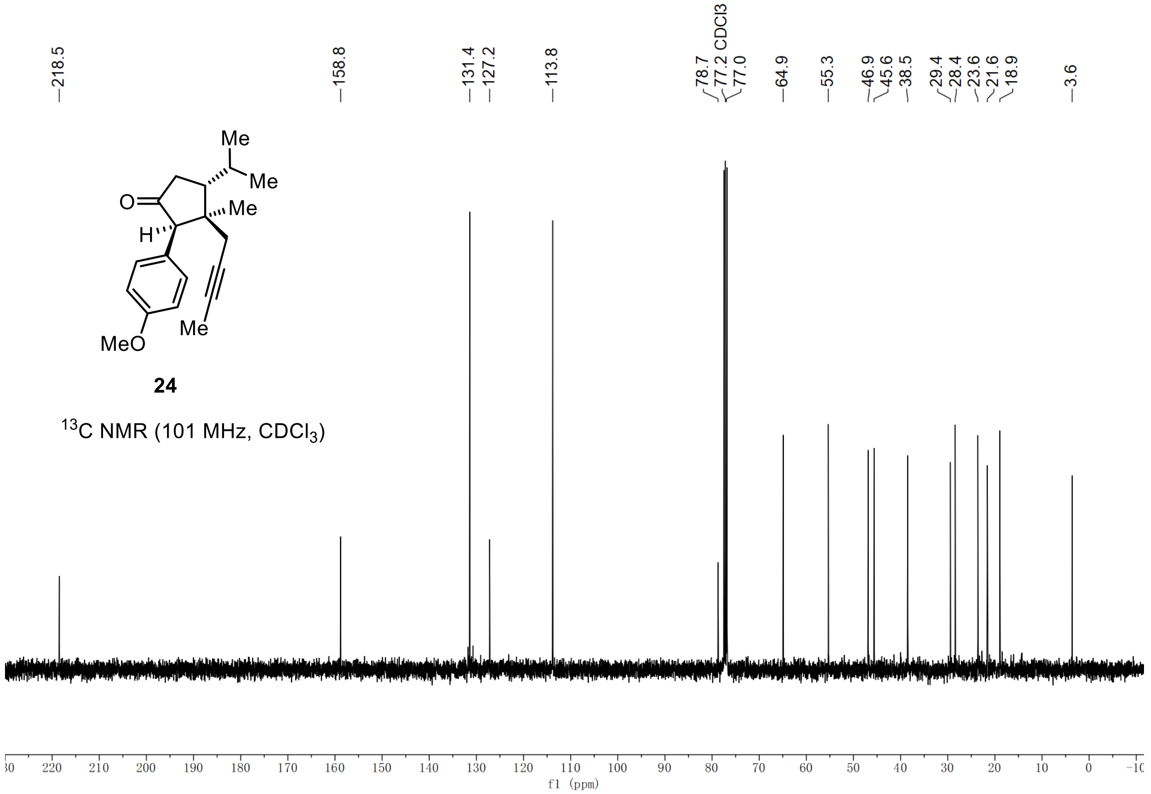

24

^{13}C NMR (101 MHz, $CDCl_3$)

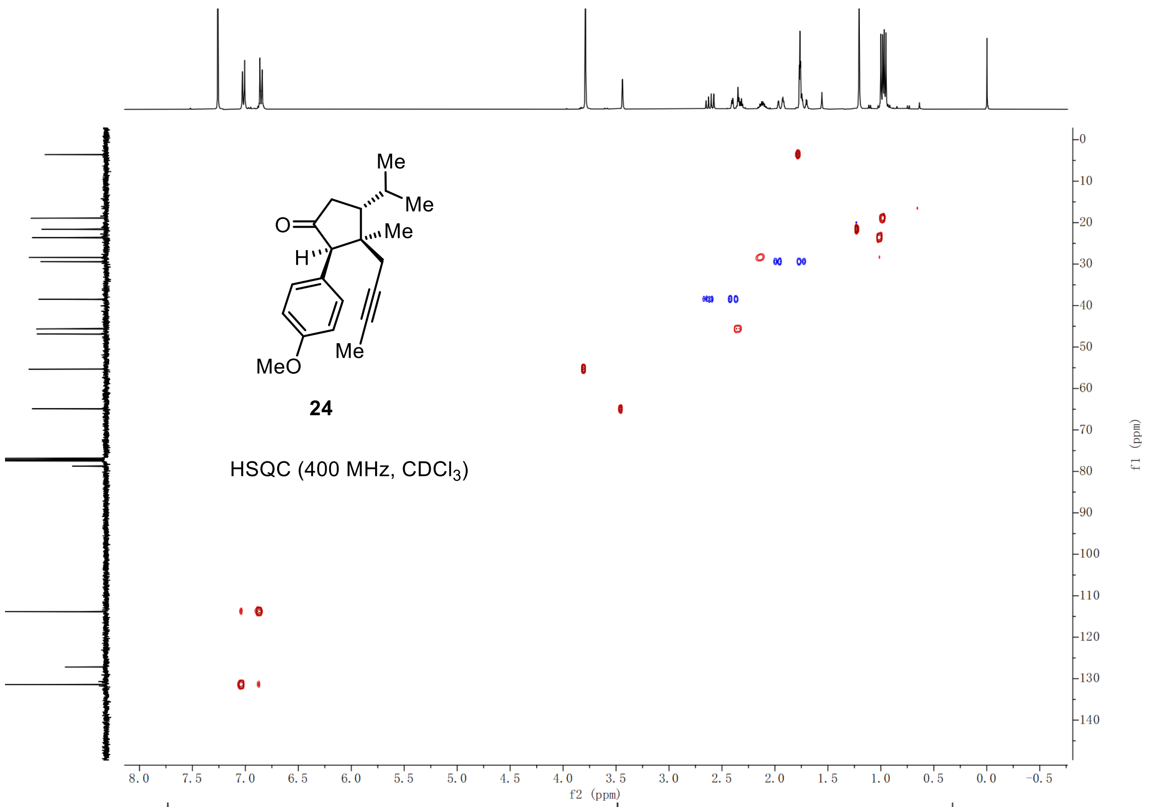

Me
Me
O
Me
H
Me
MeO
24
HSQC (400 MHz, CDCl3)
f2 (ppm)
f1 (ppm)

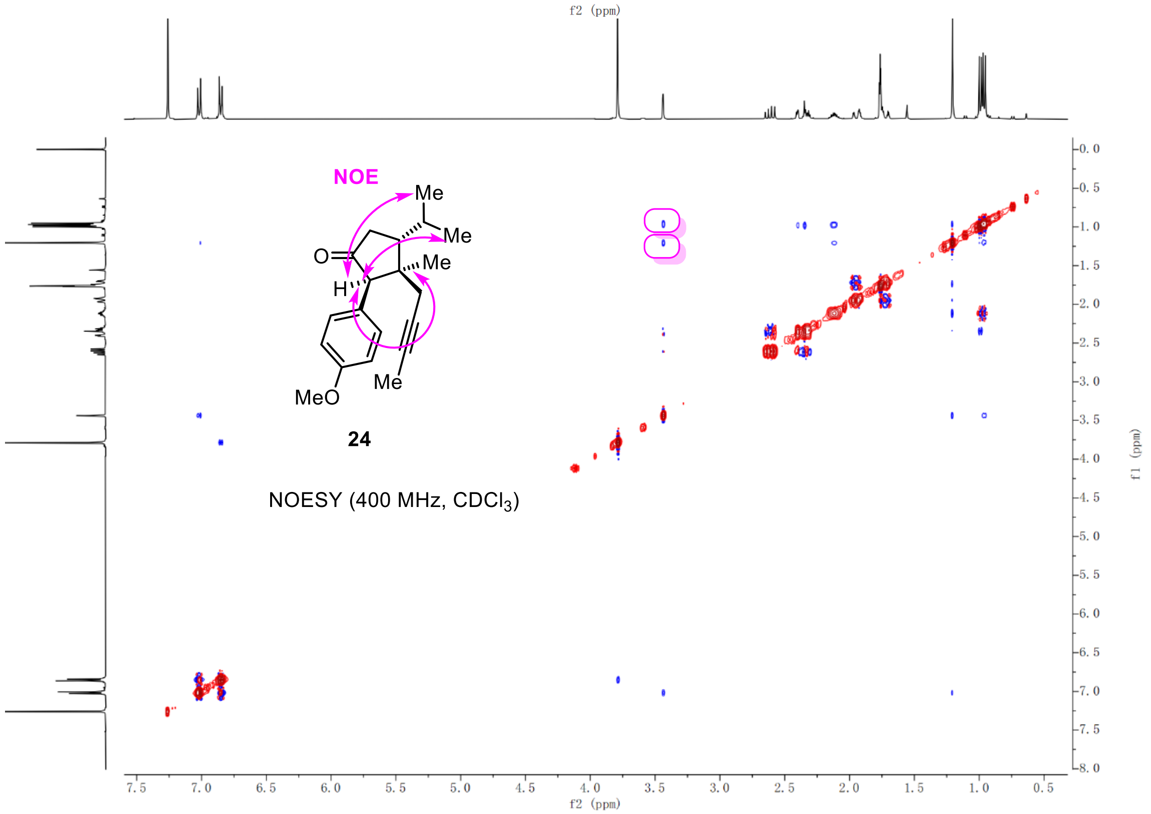

NOE
Me
Me
O
Me
H
MeO
Me
24
NOESY (400 MHz, CDCl3)
f2 (ppm)
f1 (ppm)

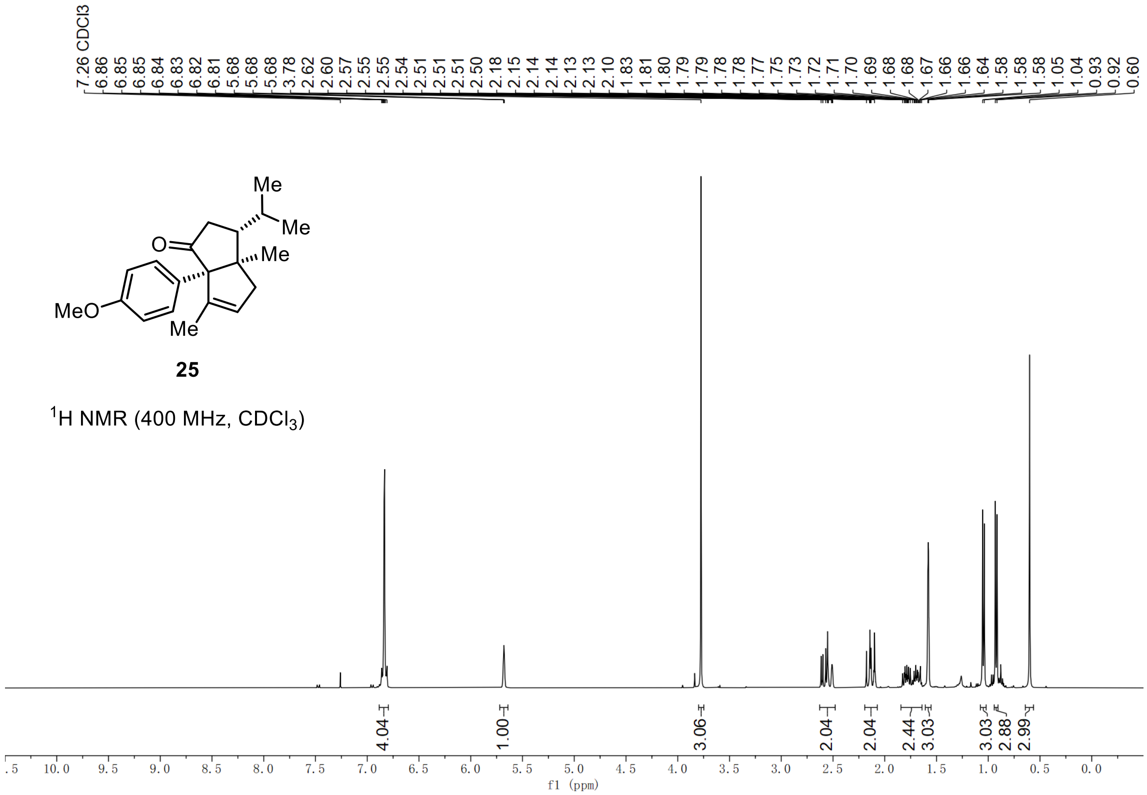
7.26 CDCl3
6.86 6.85 6.85 6.84 6.83 6.82 6.81 5.68 5.68 5.68 5.68 3.78 2.62 2.60 2.57 2.55 2.55 2.54 2.51 2.51 2.51 2.50 2.18 2.15 2.14 2.14 2.13 2.13 2.10 1.83 1.81 1.80 1.79 1.79 1.78 1.78 1.77 1.75 1.73 1.72 1.71 1.70 1.69 1.68 1.68 1.67 1.66 1.66 1.64 1.58 1.58 1.58 1.05 1.04 0.93 0.92 0.60
Me
O
Me
Me
MeO
Me
25
1H NMR (400 MHz, CDCl3)
4.04 1.00 3.06 2.04 2.04 2.44 3.03 3.03 2.88 2.99
f1 (ppm)

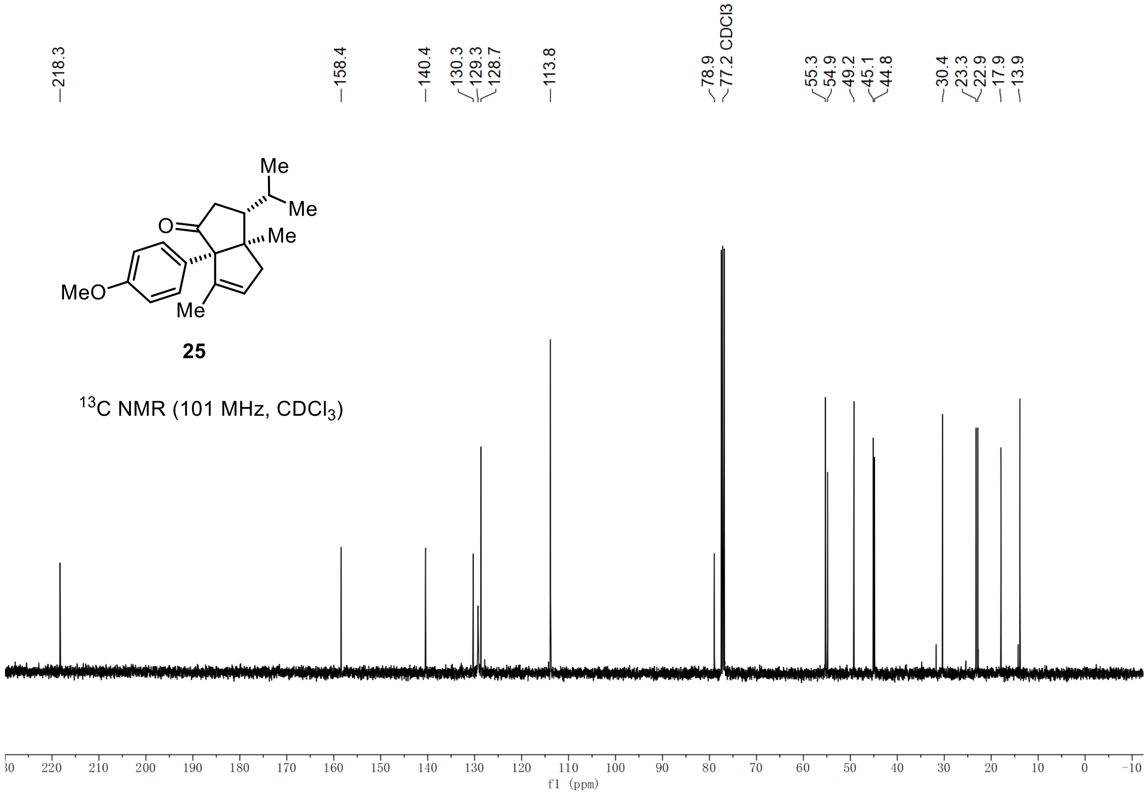
218.3
158.4
140.4
130.3 129.3 128.7
113.8
78.9 77.2 CDCl3
55.3 54.9 49.2 45.1 44.8
30.4 23.3 22.9 17.9 13.9
Me
O
Me
Me
MeO
Me
25
13C NMR (101 MHz, CDCl3)
f1 (ppm)

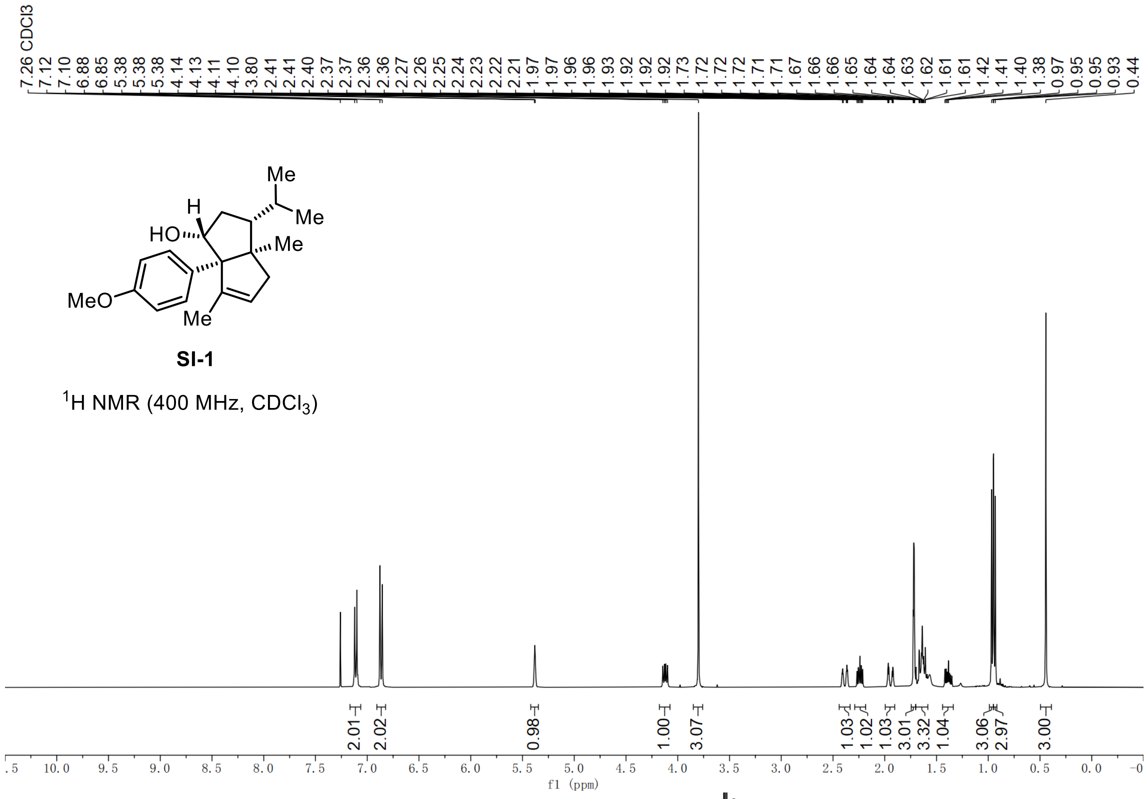

SI-1
^{1}H NMR (400 MHz, CDCl$_3$)
7.26 CDCl3
f1 (ppm)

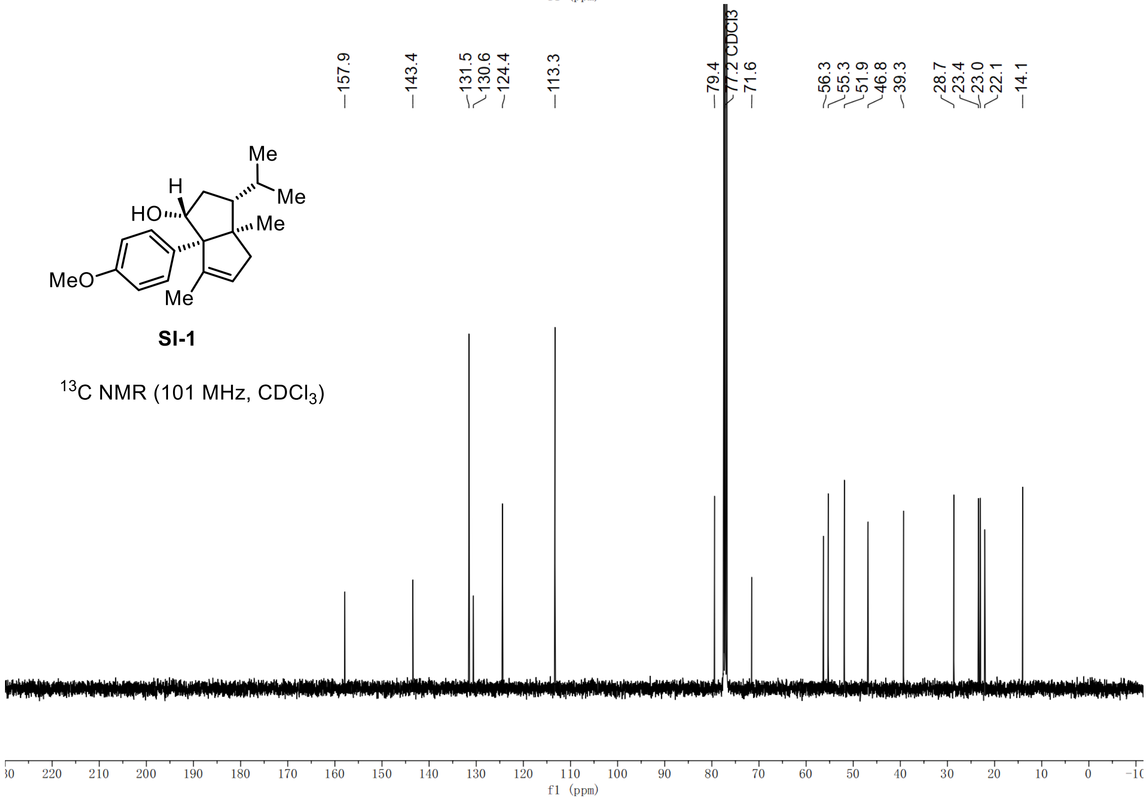

SI-1
^{13}C NMR (101 MHz, CDCl$_3$)
157.9
143.4
131.5
130.6
124.4
113.3
79.4
77.2 CDCl3
71.6
56.3
55.3
51.9
46.8
39.3
28.7
23.4
23.0
22.1
14.1
f1 (ppm)

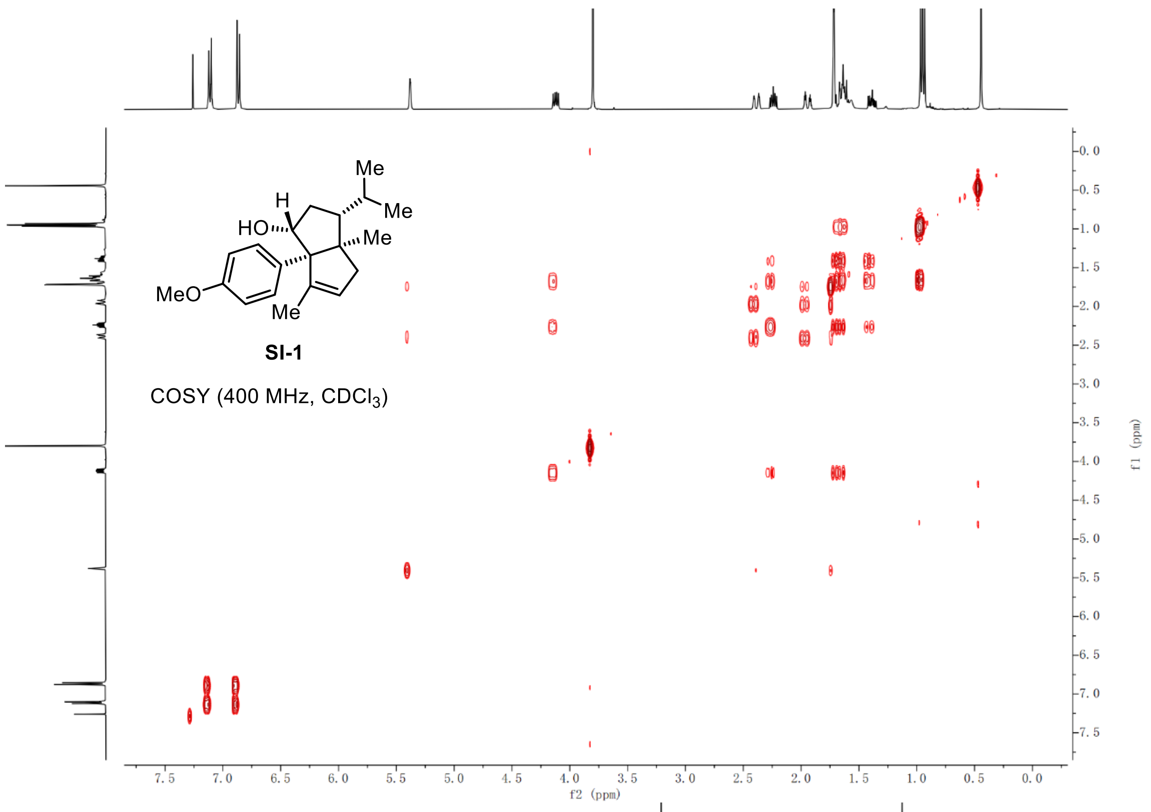
Me
H
Me
HO
Me
MeO
Me
SI-1
COSY (400 MHz, CDCl3)
f2 (ppm)
f1 (ppm)

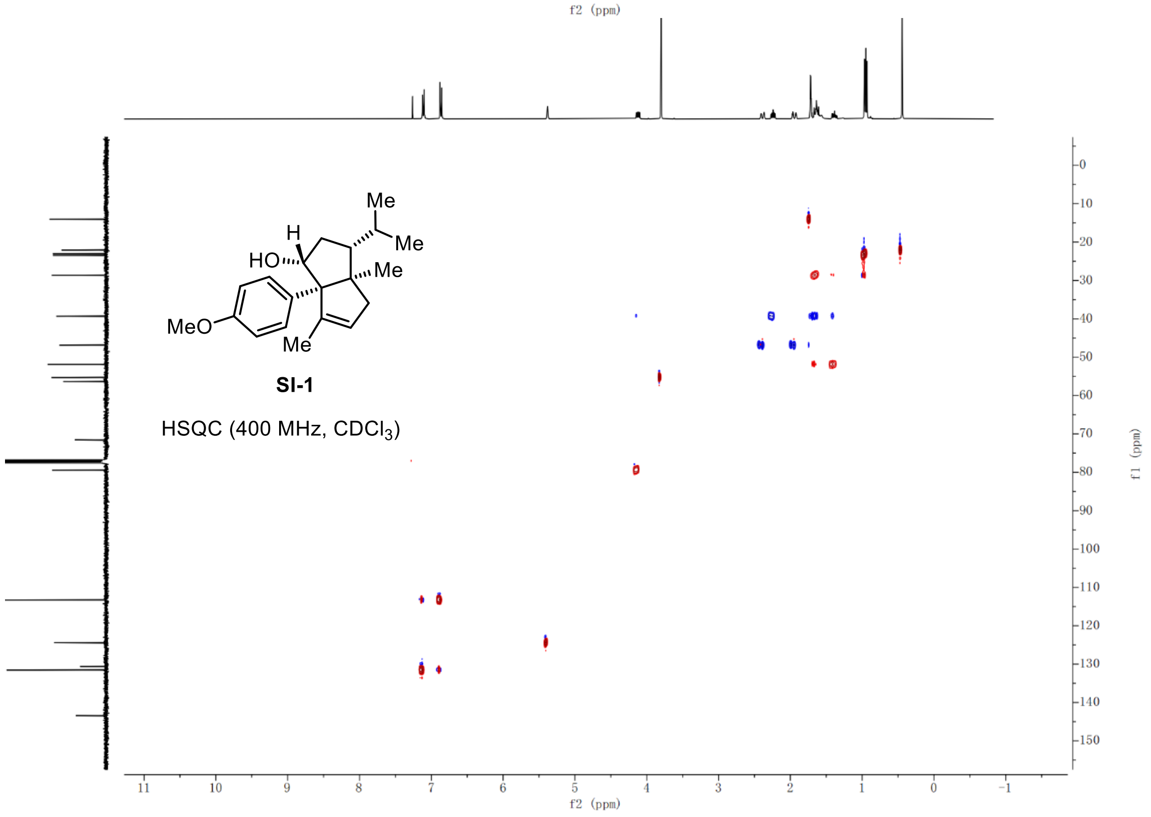
Me
H
Me
HO
Me
MeO
Me
SI-1
HSQC (400 MHz, CDCl3)
f2 (ppm)
f1 (ppm)

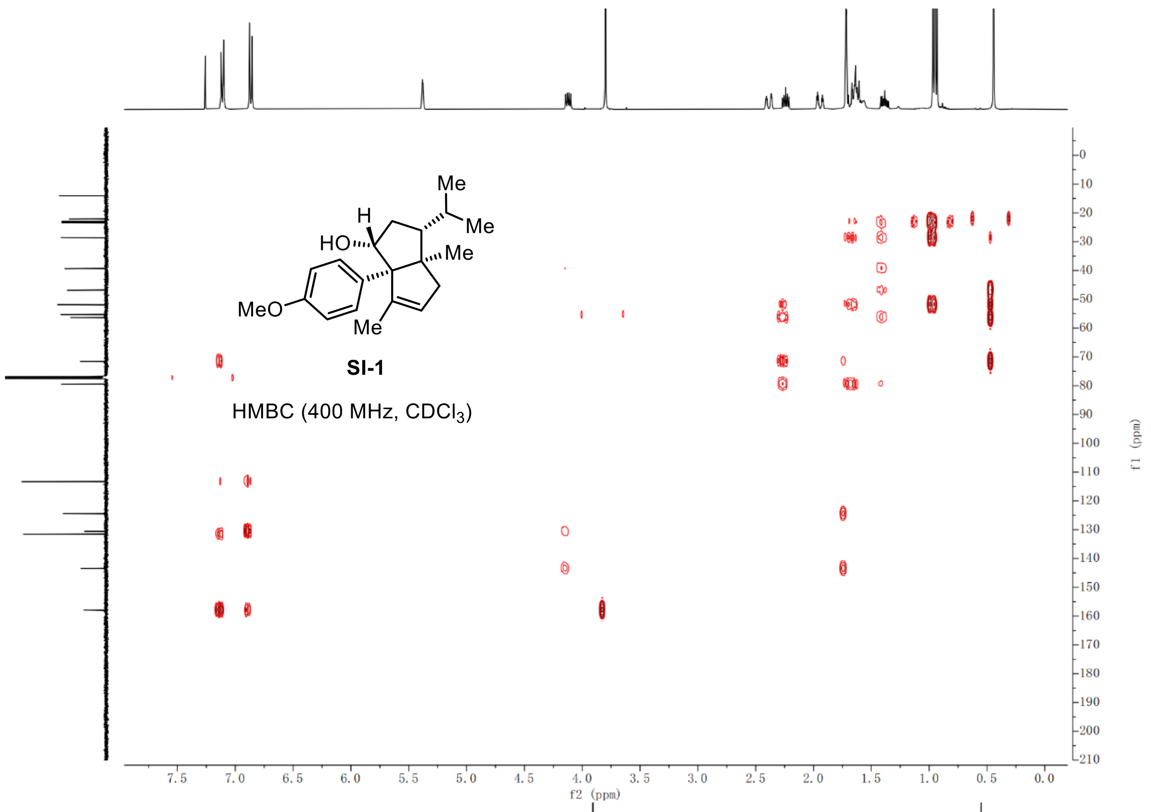
Me
H
Me
HO
Me
MeO
Me
SI-1
HMBC (400 MHz, CDCl3)
f2 (ppm)
f1 (ppm)

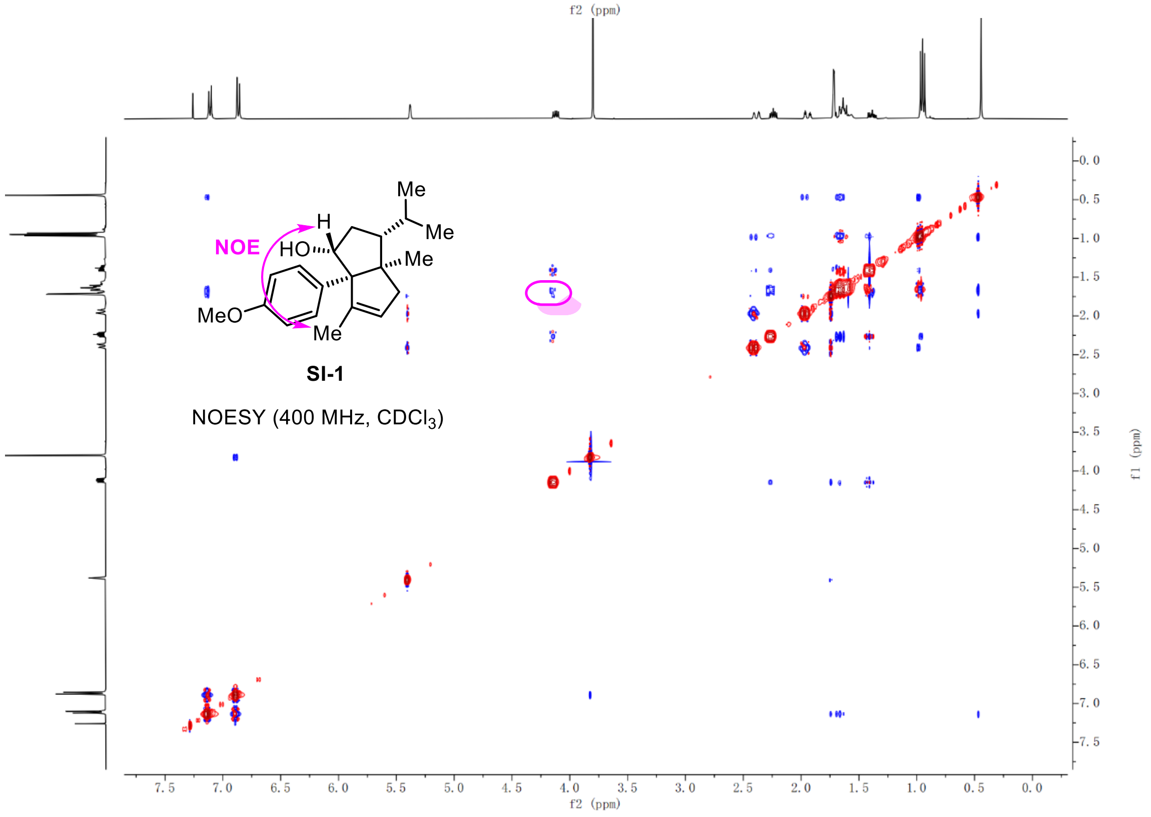
Me
H
Me
NOE
HO
Me
MeO
Me
SI-1
NOESY (400 MHz, CDCl3)
f2 (ppm)
f1 (ppm)

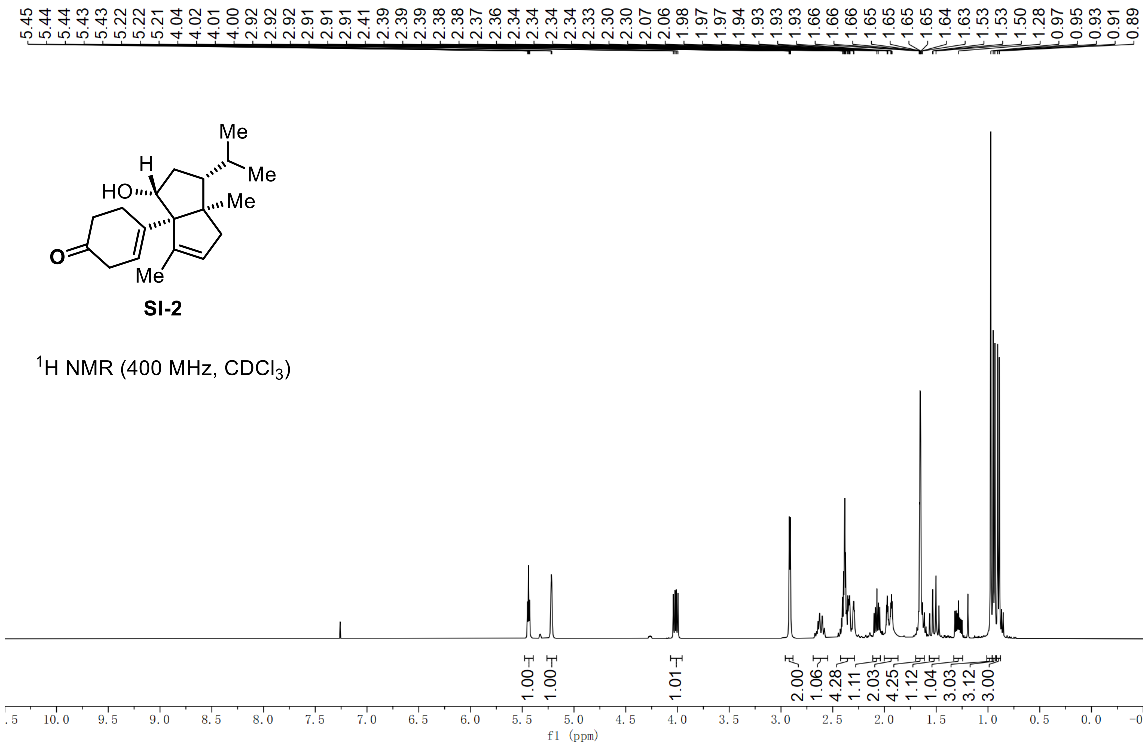
5.45 5.44 5.44 5.43 5.22 5.22 5.21 4.04 4.02 4.01 4.00 2.92 2.92 2.92 2.91 2.91 2.91 2.41 2.39 2.39 2.39 2.38 2.38 2.37 2.36 2.34 2.34 2.34 2.34 2.33 2.30 2.30 2.07 2.06 1.98 1.97 1.97 1.94 1.93 1.93 1.93 1.66 1.66 1.66 1.65 1.65 1.65 1.64 1.63 1.53 1.53 1.50 1.28 0.97 0.95 0.93 0.91 0.89
Me
H
Me
HO
Me
O
Me
SI-2
1H NMR (400 MHz, CDCl3)
1.00 1.00 1.01 2.00 1.06 4.28 1.11 2.03 4.25 1.12 1.04 3.03 3.12 3.00
f1 (ppm)

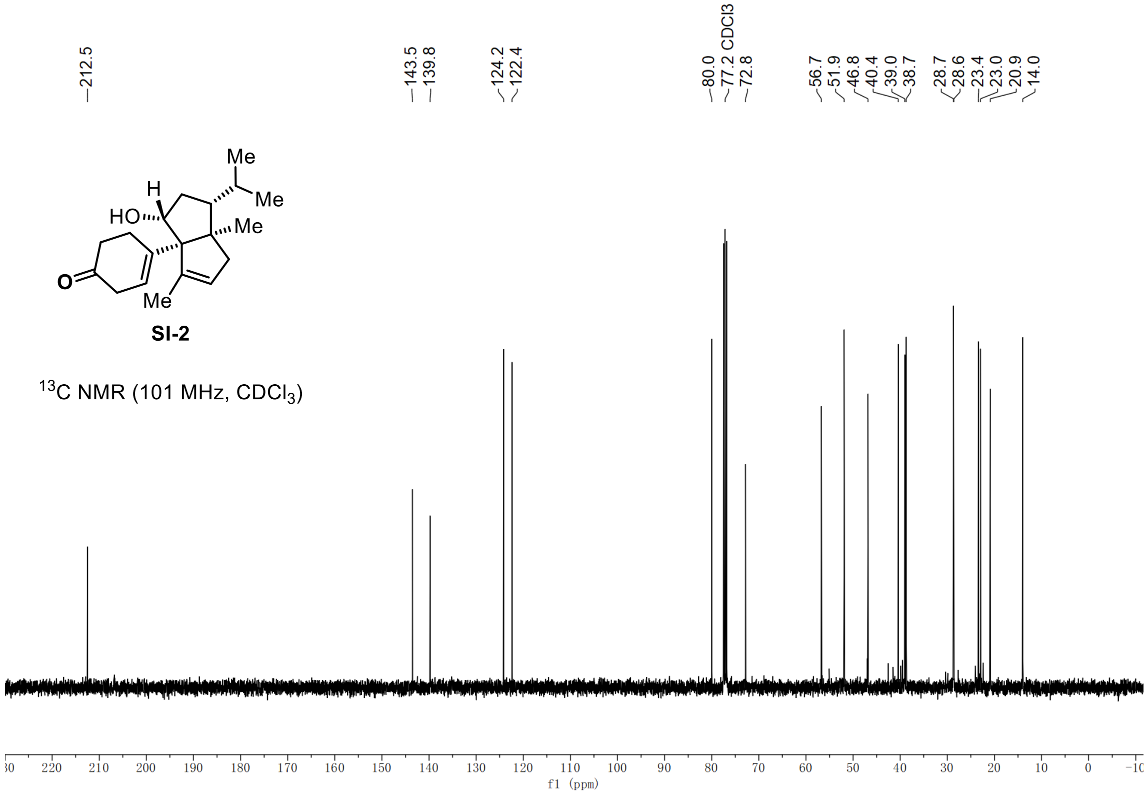
212.5
143.5 139.8
124.2 122.4
80.0 77.2 CDCl3 72.8
56.7 51.9 46.8 40.4 39.0 38.7
28.7 28.6 23.4 23.0 20.9 14.0
Me
H
Me
HO
Me
O
Me
SI-2
13C NMR (101 MHz, CDCl3)
f1 (ppm)

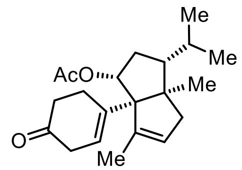

26

^{1}H NMR (400 MHz, $CDCl_3$)

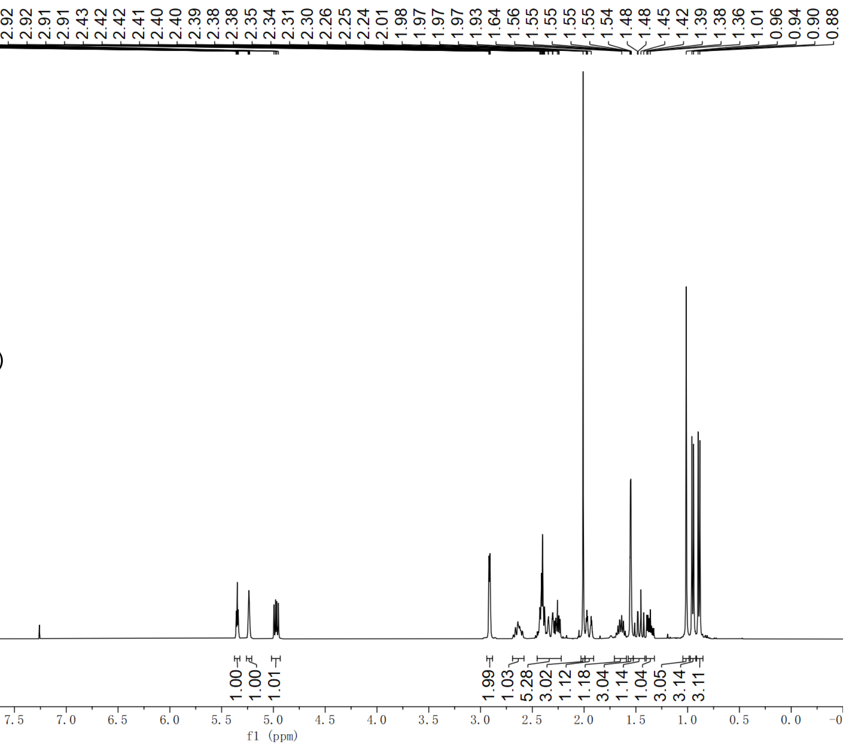

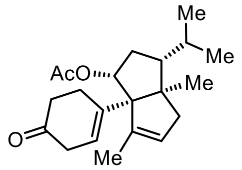

26

^{13}C NMR (101 MHz, $CDCl_3$)

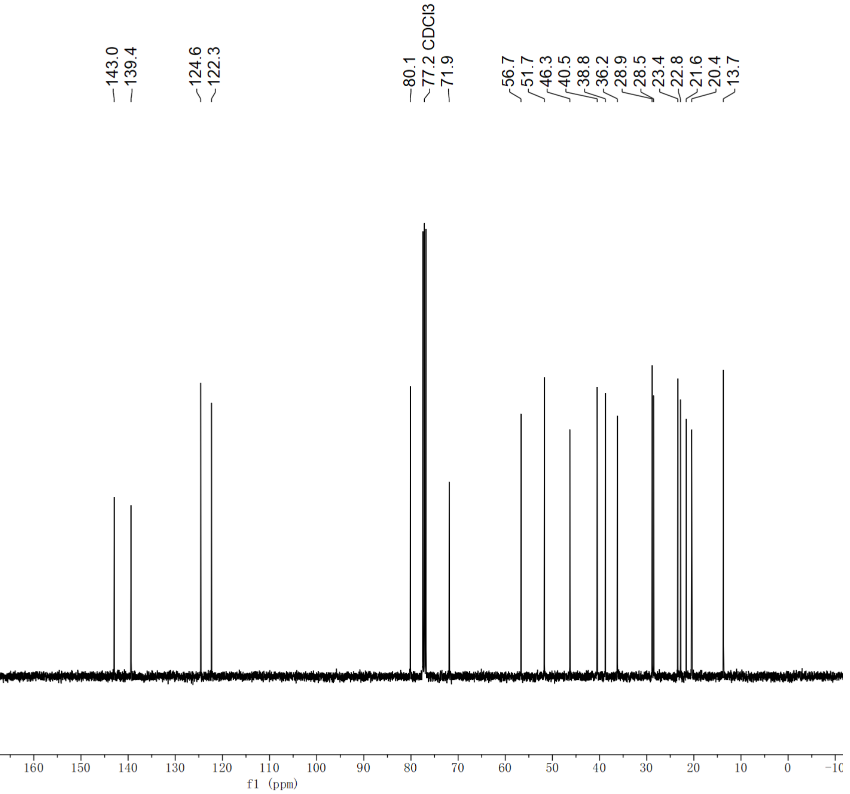

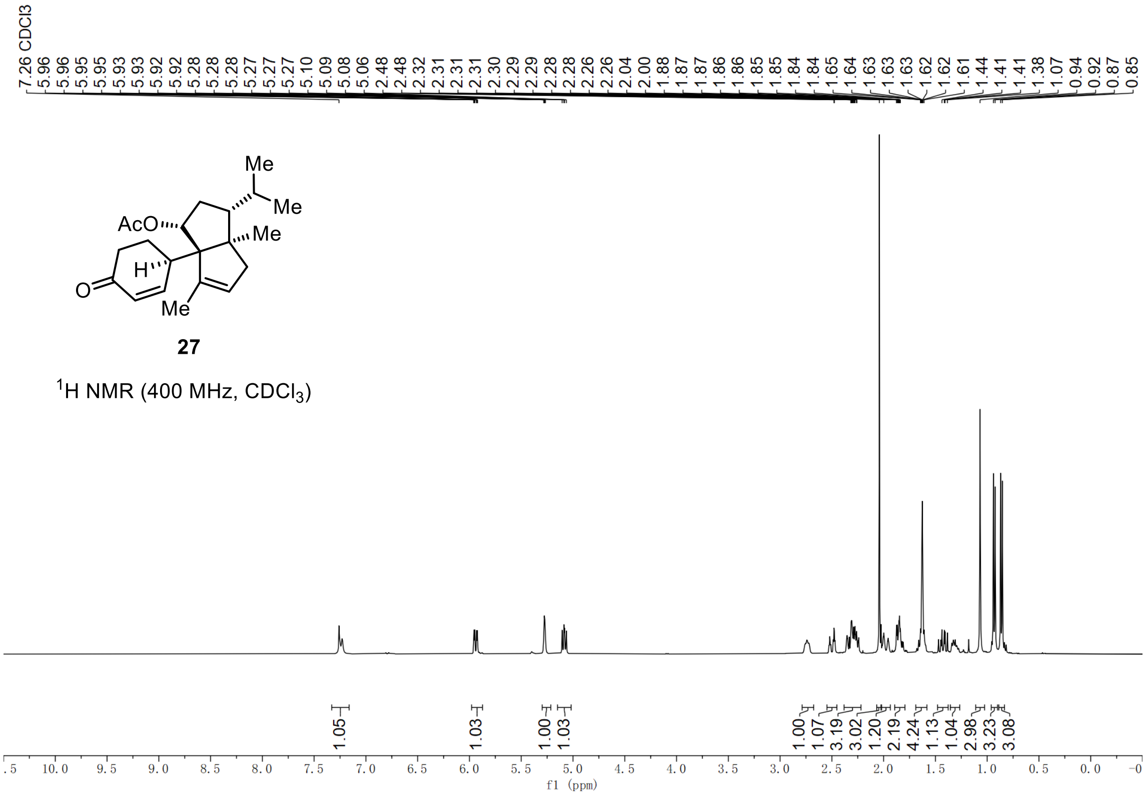
7.26 CDCl3
5.96
5.96
5.95
5.95
5.93
5.93
5.92
5.92
5.28
5.28
5.28
5.27
5.27
5.27
5.10
5.09
5.08
5.06
2.48
2.48
2.32
2.31
2.31
2.31
2.30
2.29
2.29
2.28
2.28
2.26
2.26
2.04
2.00
1.88
1.87
1.87
1.86
1.86
1.85
1.85
1.84
1.84
1.65
1.64
1.63
1.63
1.63
1.62
1.62
1.61
1.44
1.41
1.41
1.38
1.07
0.94
0.92
0.87
0.85
Me
AcO
Me
Me
H
O
Me
27
^{1}H NMR (400 MHz, $CDCl_3$)
1.05
1.03
1.00
1.03
1.00
1.07
3.19
3.02
1.20
2.19
4.24
1.13
1.04
2.98
3.23
3.08
f1 (ppm)

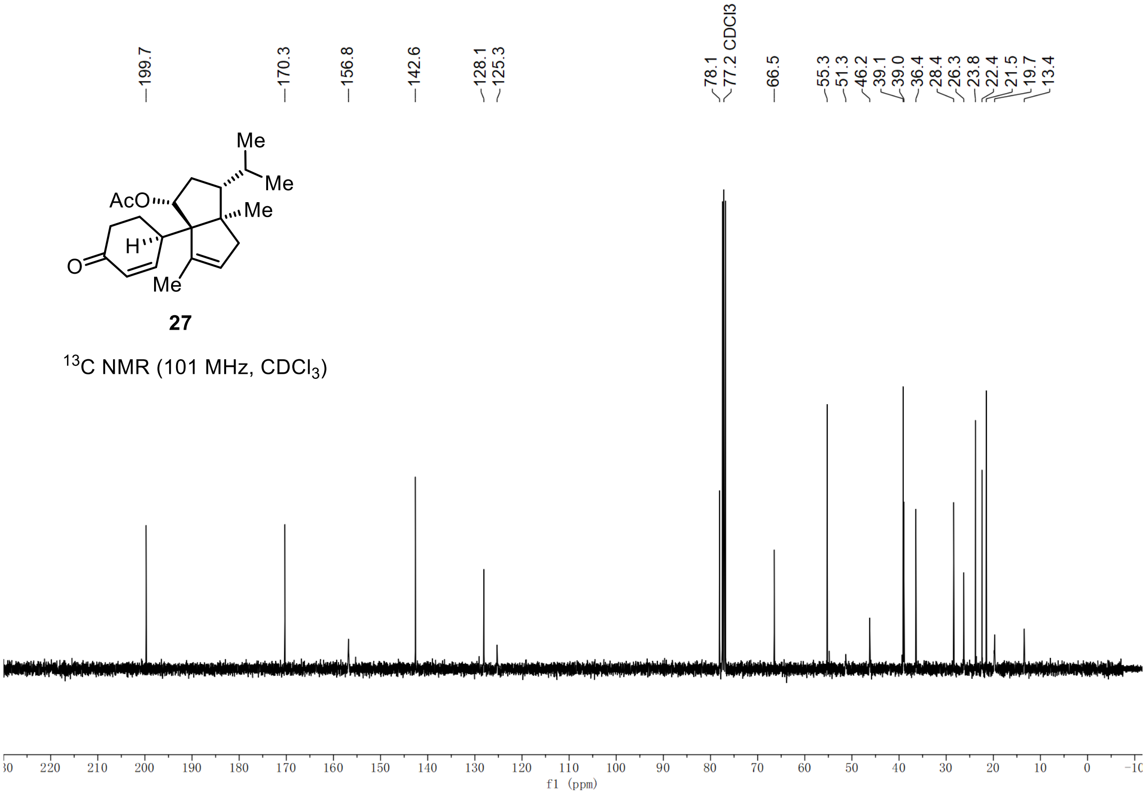
199.7
170.3
156.8
142.6
128.1
125.3
78.1
77.2 CDCl3
66.5
55.3
51.3
46.2
39.1
39.0
36.4
28.4
26.3
23.8
22.4
21.5
19.7
13.4
Me
AcO
Me
Me
H
O
Me
27
^{13}C NMR (101 MHz, $CDCl_3$)
f1 (ppm)

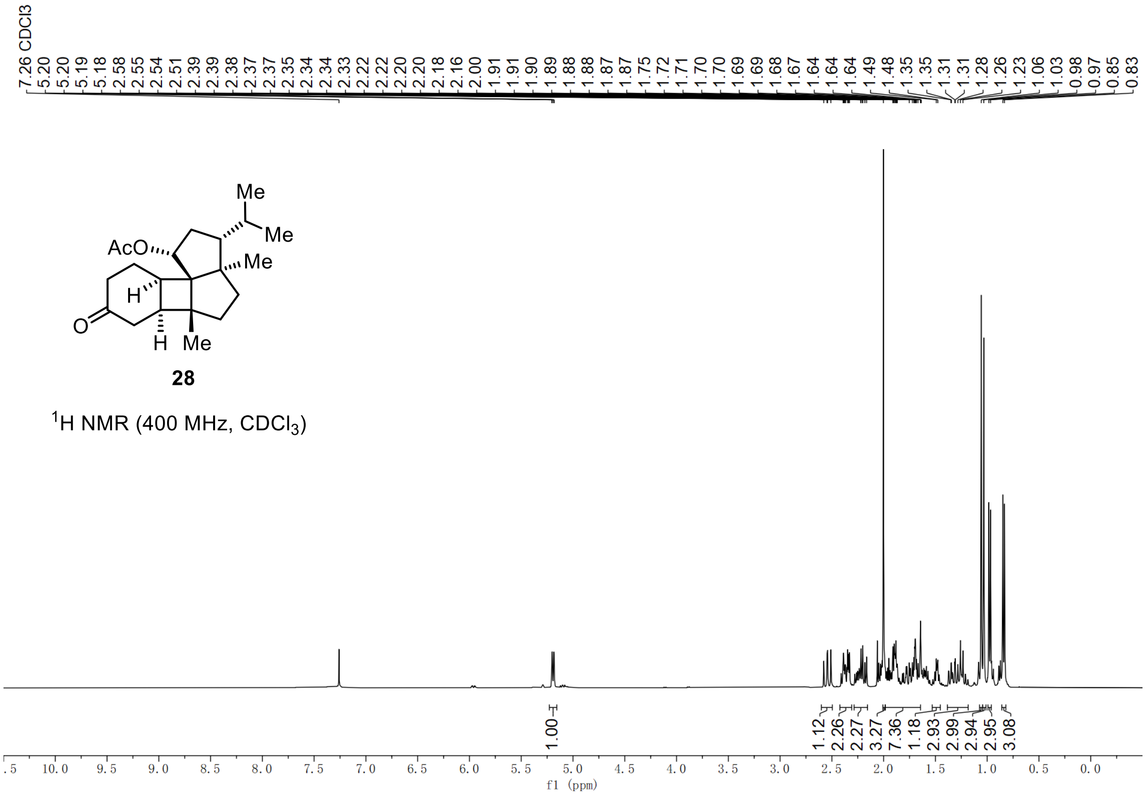

28

^{1}H NMR (400 MHz, $CDCl_3$)

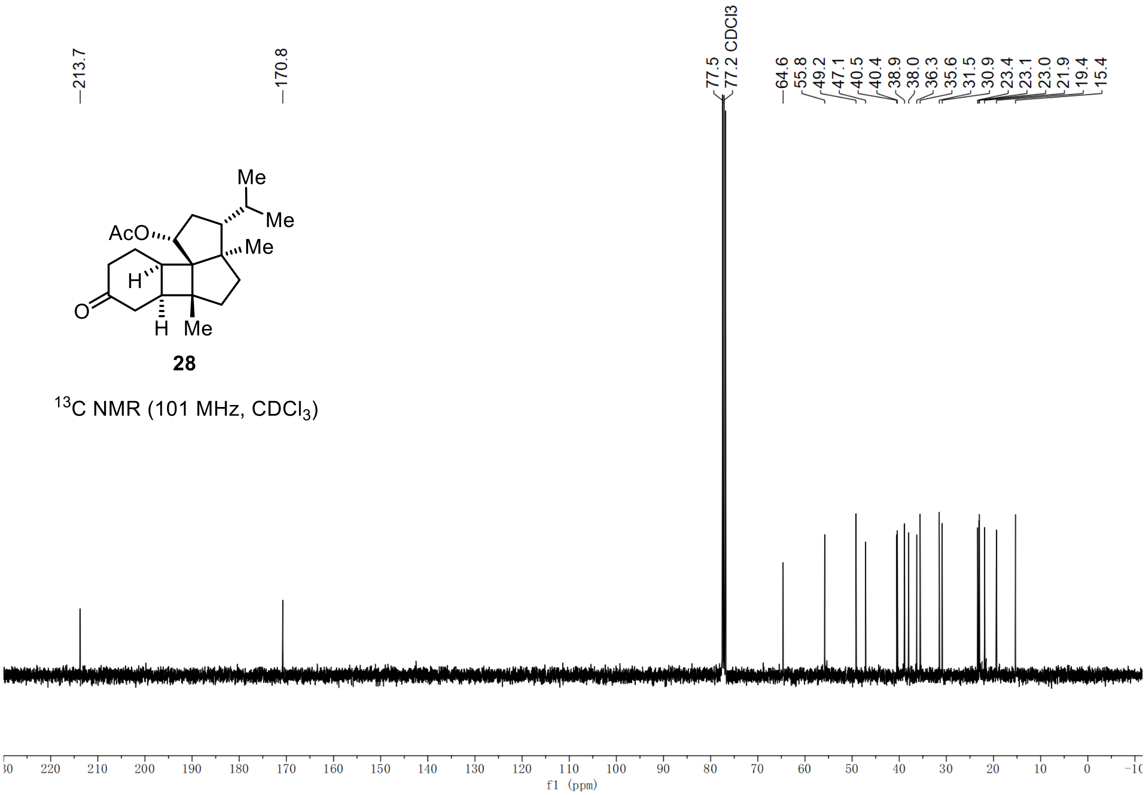

28

^{13}C NMR (101 MHz, $CDCl_3$)

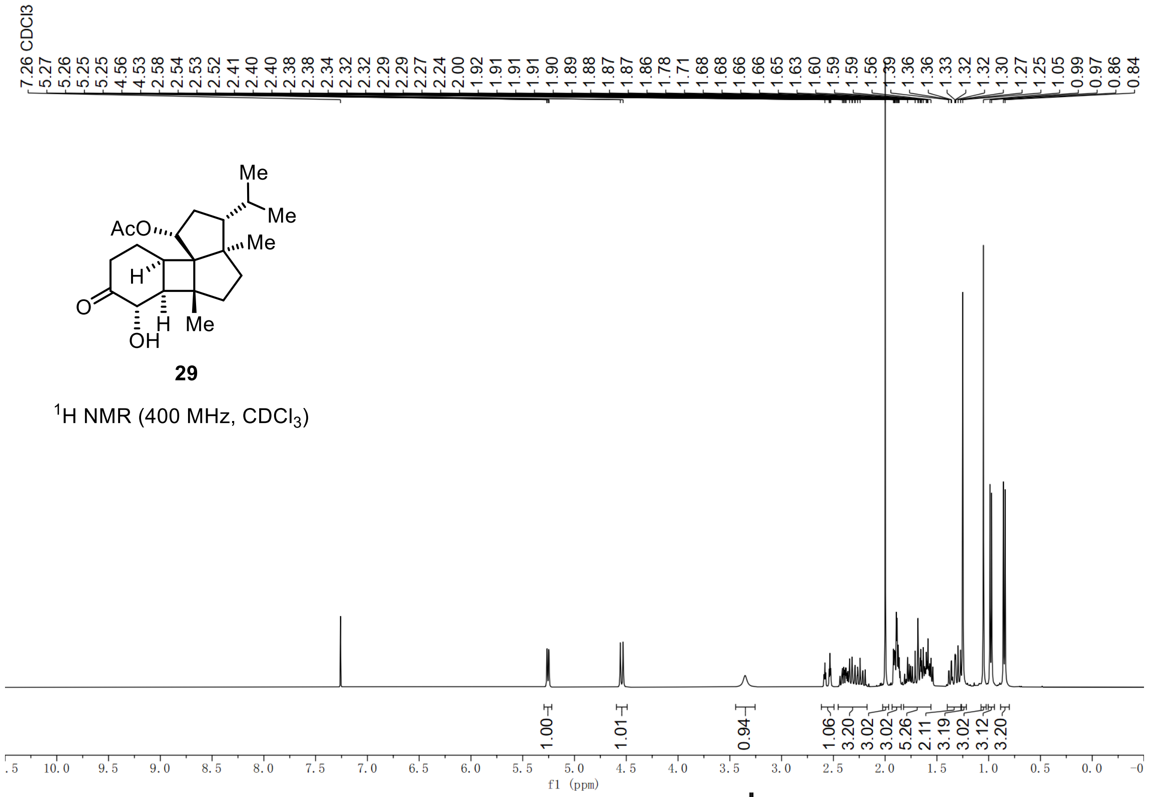

29

^1H NMR (400 MHz, $CDCl_3$)

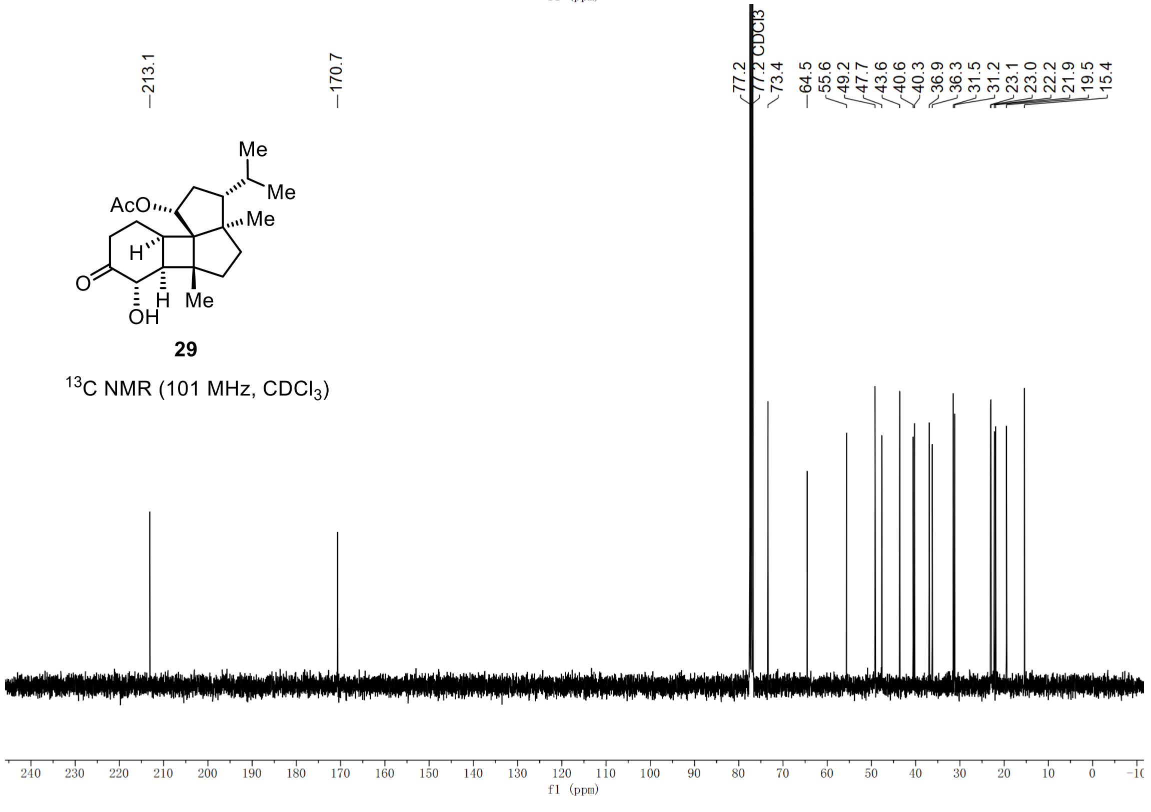

29

^{13}C NMR (101 MHz, $CDCl_3$)

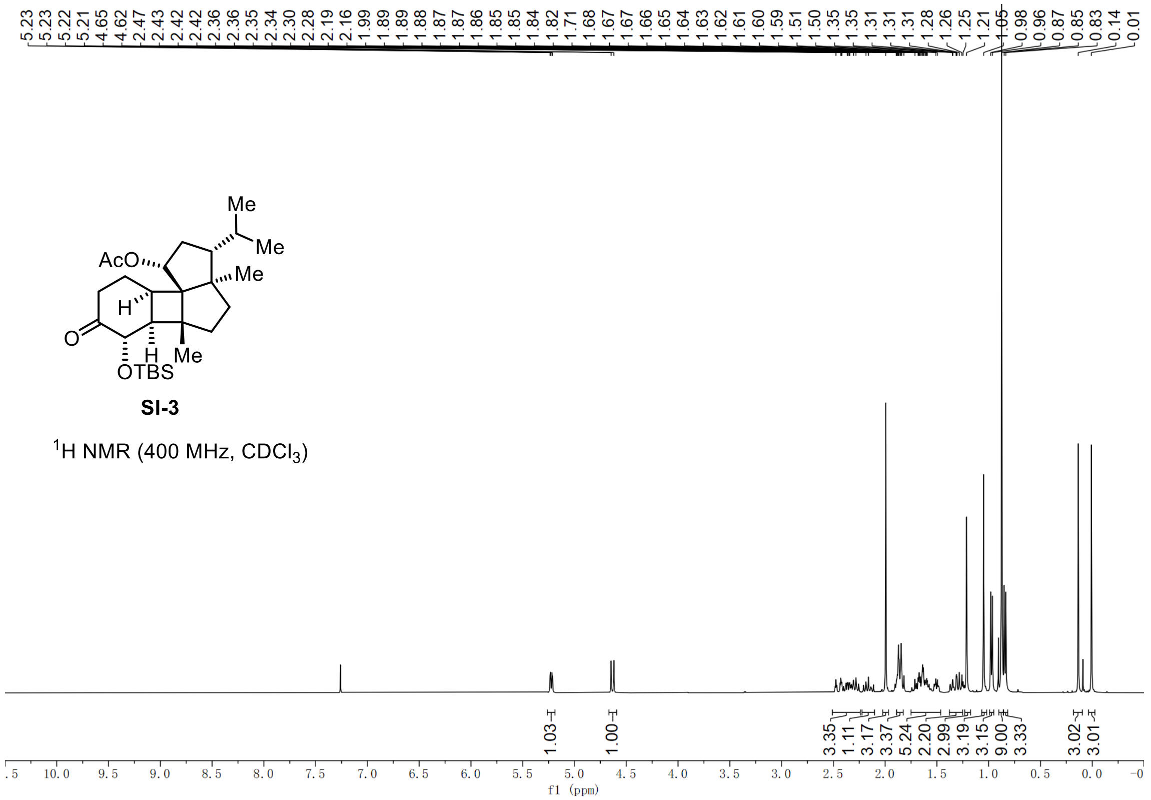

SI-3
^{1}H NMR (400 MHz, CDCl$_3$)
f1 (ppm)

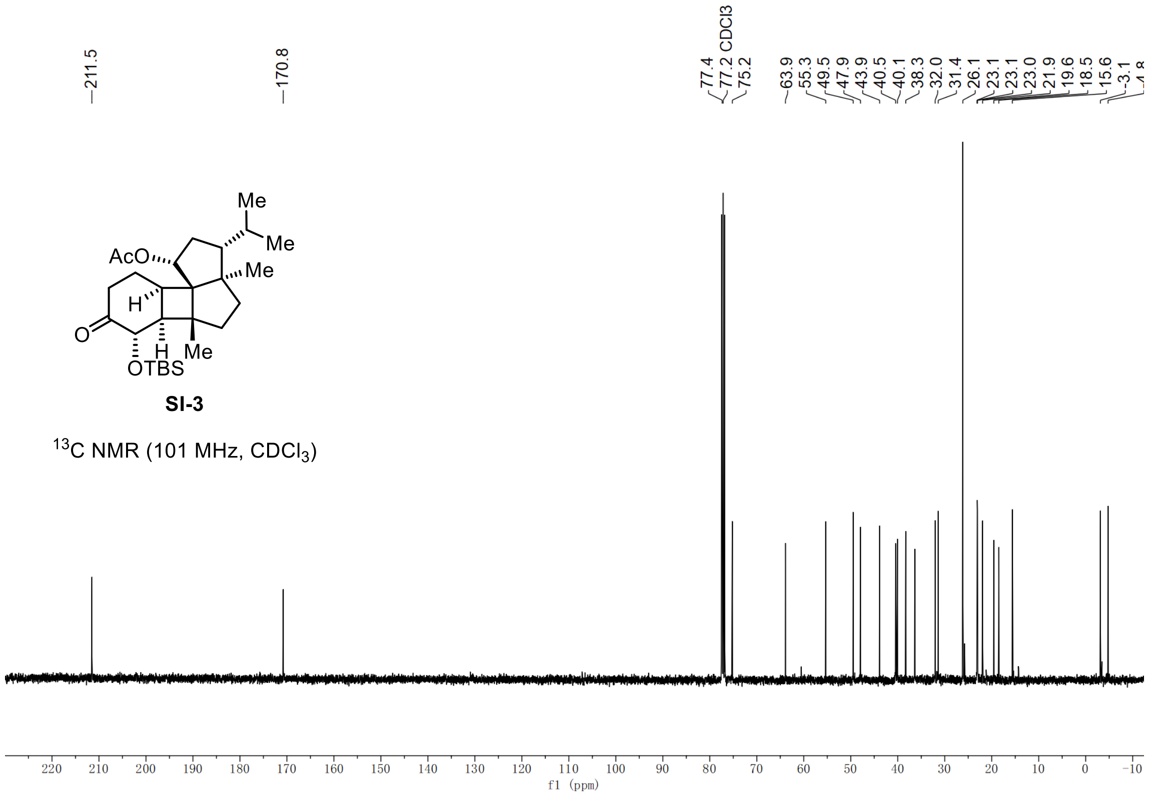

211.5
170.8
77.4
77.2 CDCl3
75.2
63.9
55.3
49.5
47.9
43.9
40.5
40.1
38.3
32.0
31.4
26.1
23.1
23.1
23.0
21.9
19.6
18.5
15.6
-3.1
SI-3
^{13}C NMR (101 MHz, CDCl$_3$)
f1 (ppm)

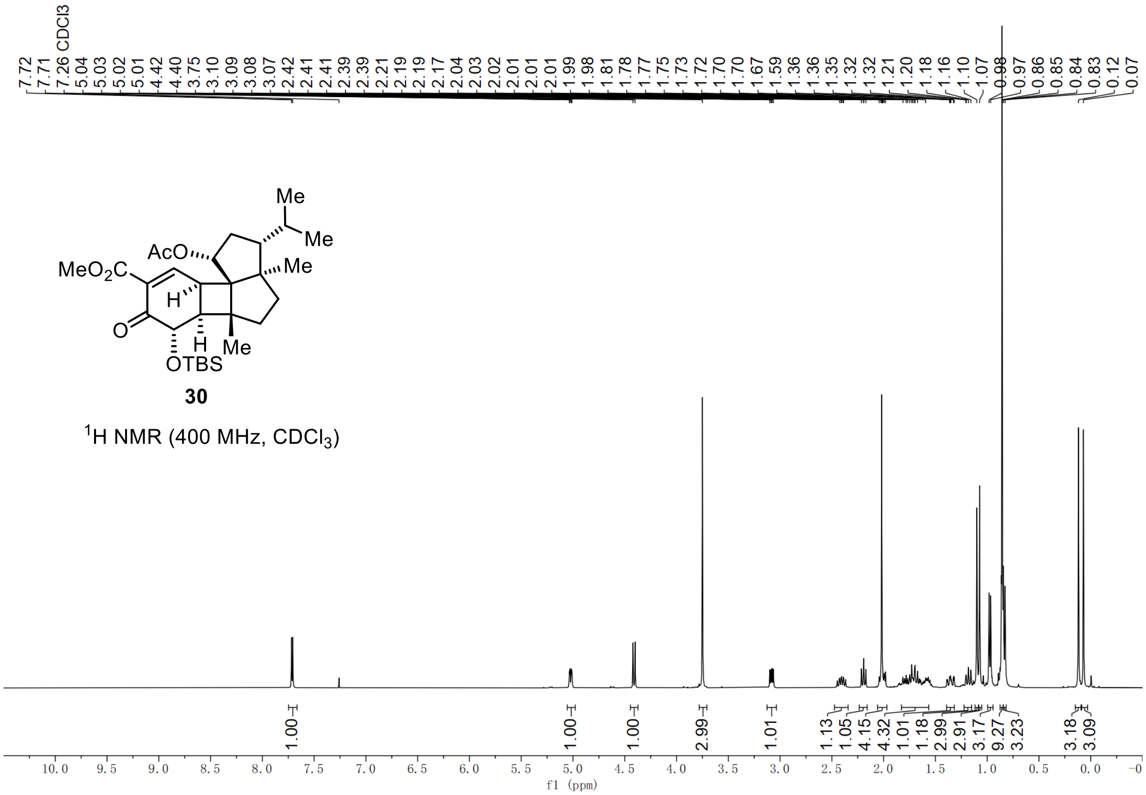

30

^{1}H NMR (400 MHz, $CDCl_3$)

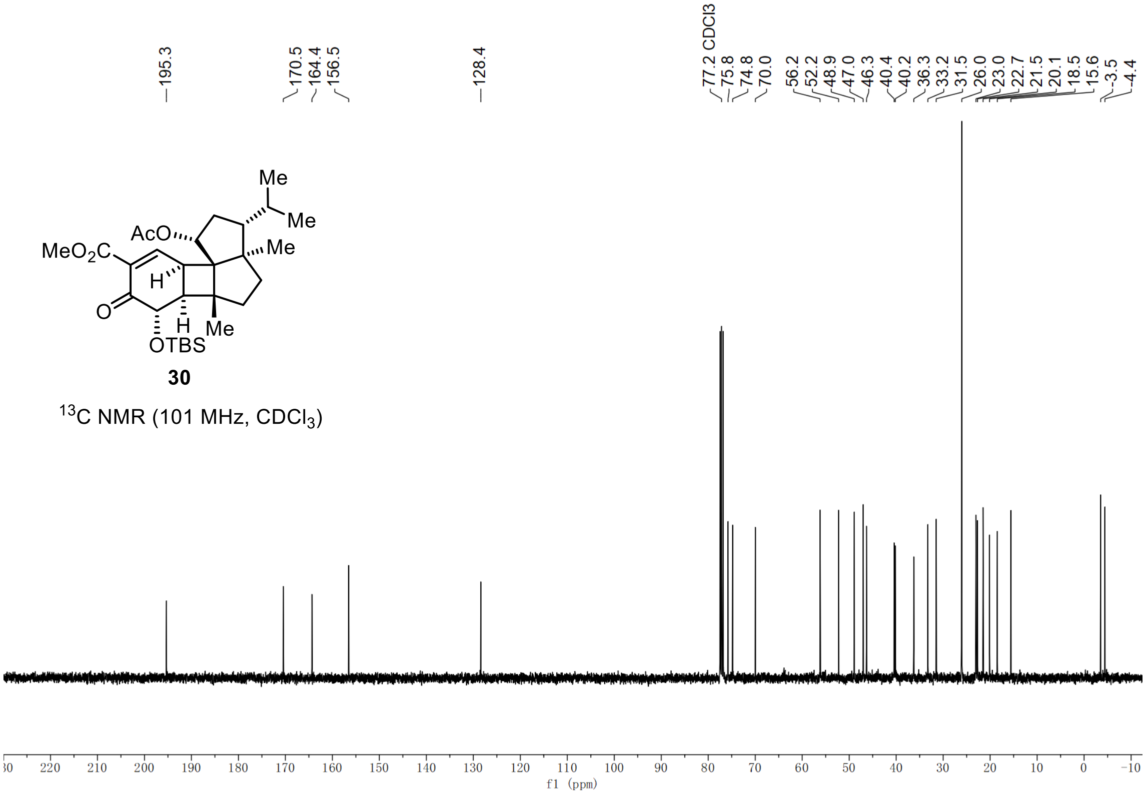

30

^{13}C NMR (101 MHz, $CDCl_3$)

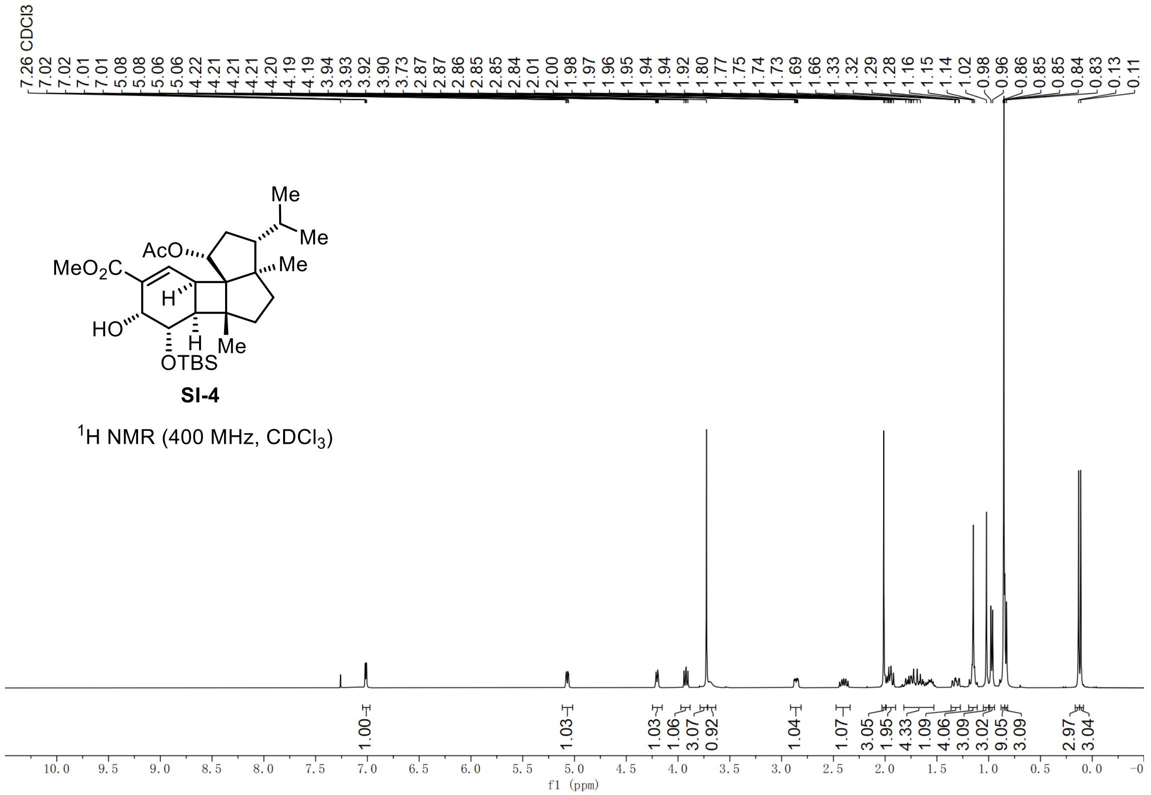
7.26 CDCl3
7.02
7.02
7.01
7.01
5.08
5.08
5.06
5.06
4.22
4.21
4.21
4.21
4.20
4.19
4.19
3.94
3.93
3.92
3.90
3.73
2.87
2.87
2.86
2.85
2.85
2.84
2.01
2.00
1.98
1.97
1.96
1.95
1.94
1.94
1.92
1.80
1.77
1.75
1.74
1.73
1.69
1.66
1.33
1.32
1.29
1.28
1.16
1.15
1.14
1.02
0.98
0.96
0.86
0.85
0.85
0.84
0.83
0.13
0.11
Me
Me
AcO
Me
MeO2C
H
HO
H
Me
OTBS
SI-4
1H NMR (400 MHz, CDCl3)
1.00
1.03
1.03
1.06
3.07
0.92
1.04
1.07
3.05
1.95
4.33
1.09
4.06
3.09
3.02
9.05
3.09
2.97
3.04
10.0 9.5 9.0 8.5 8.0 7.5 7.0 6.5 6.0 5.5 5.0 4.5 4.0 3.5 3.0 2.5 2.0 1.5 1.0 0.5 0.0
f1 (ppm)

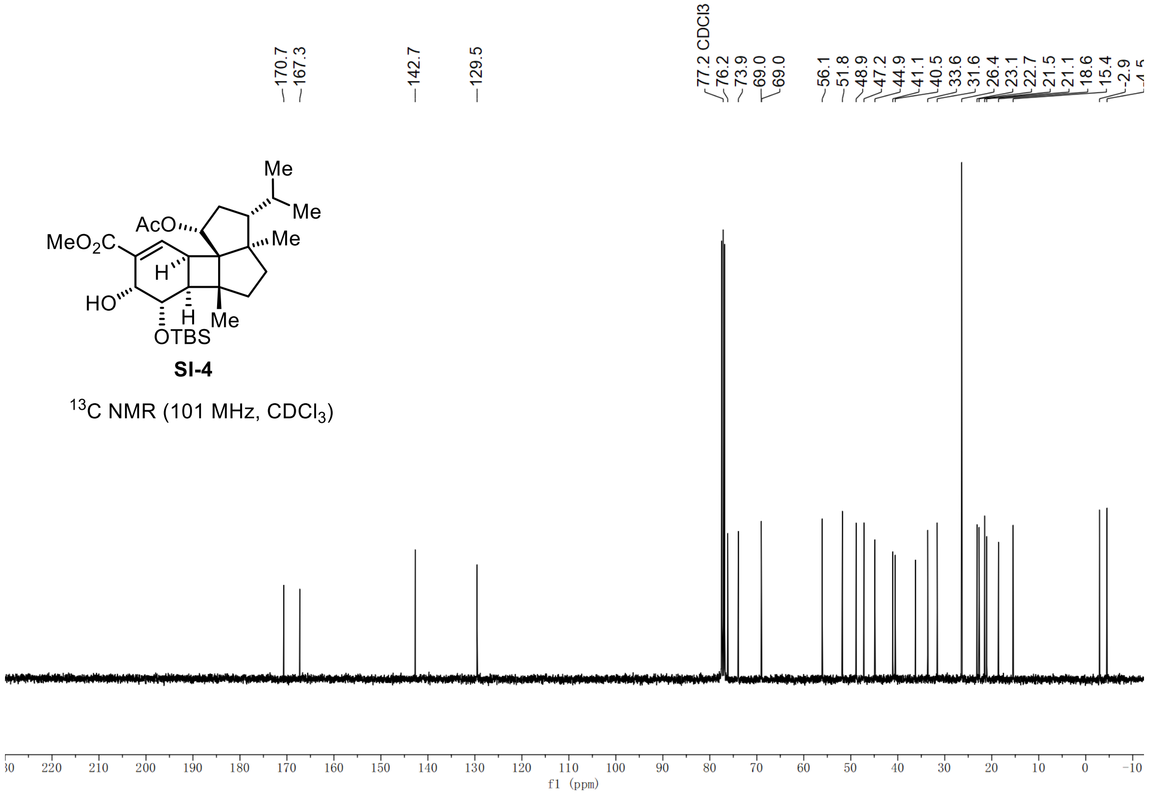
170.7
167.3
142.7
129.5
77.2 CDCl3
76.2
73.9
69.0
69.0
56.1
51.8
48.9
47.2
44.9
41.1
40.5
33.6
31.6
26.4
23.1
22.7
21.5
21.1
18.6
15.4
-2.9
Me
Me
AcO
Me
MeO2C
H
HO
H
Me
OTBS
SI-4
13C NMR (101 MHz, CDCl3)
220 210 200 190 180 170 160 150 140 130 120 110 100 90 80 70 60 50 40 30 20 10 0 -10
f1 (ppm)

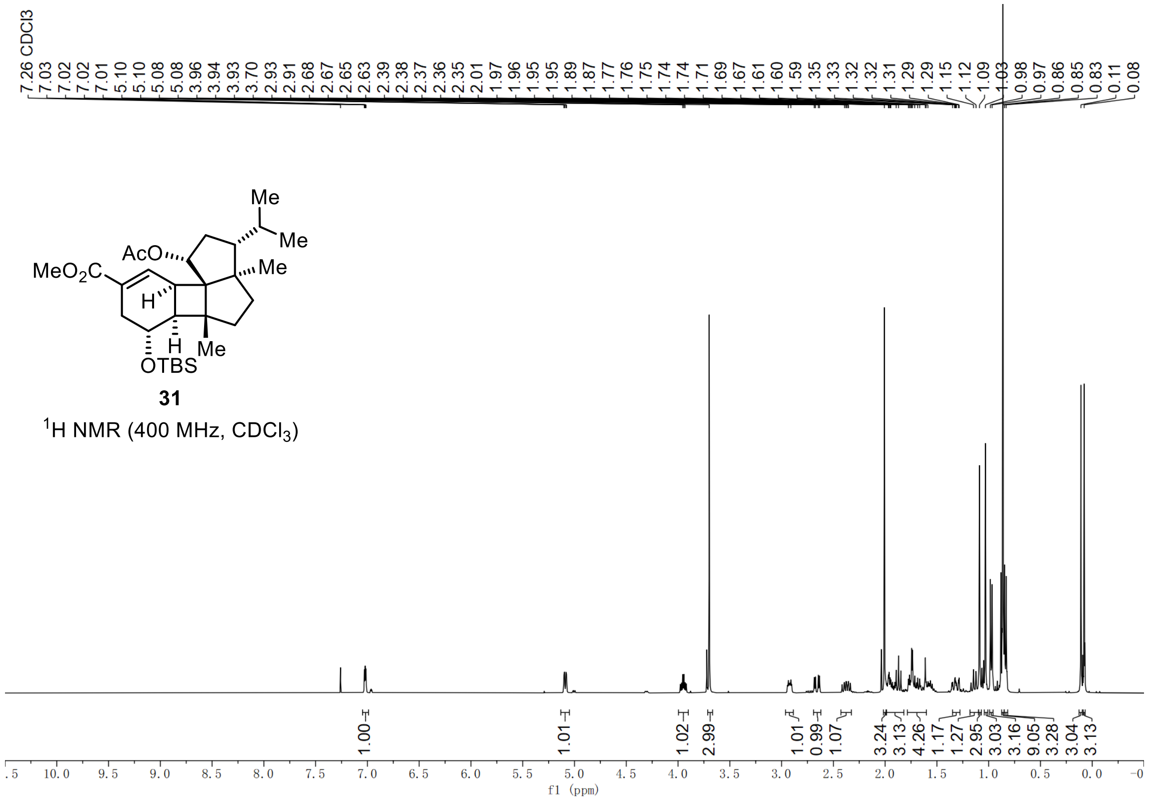

31

^{1}H NMR (400 MHz, $CDCl_3$)

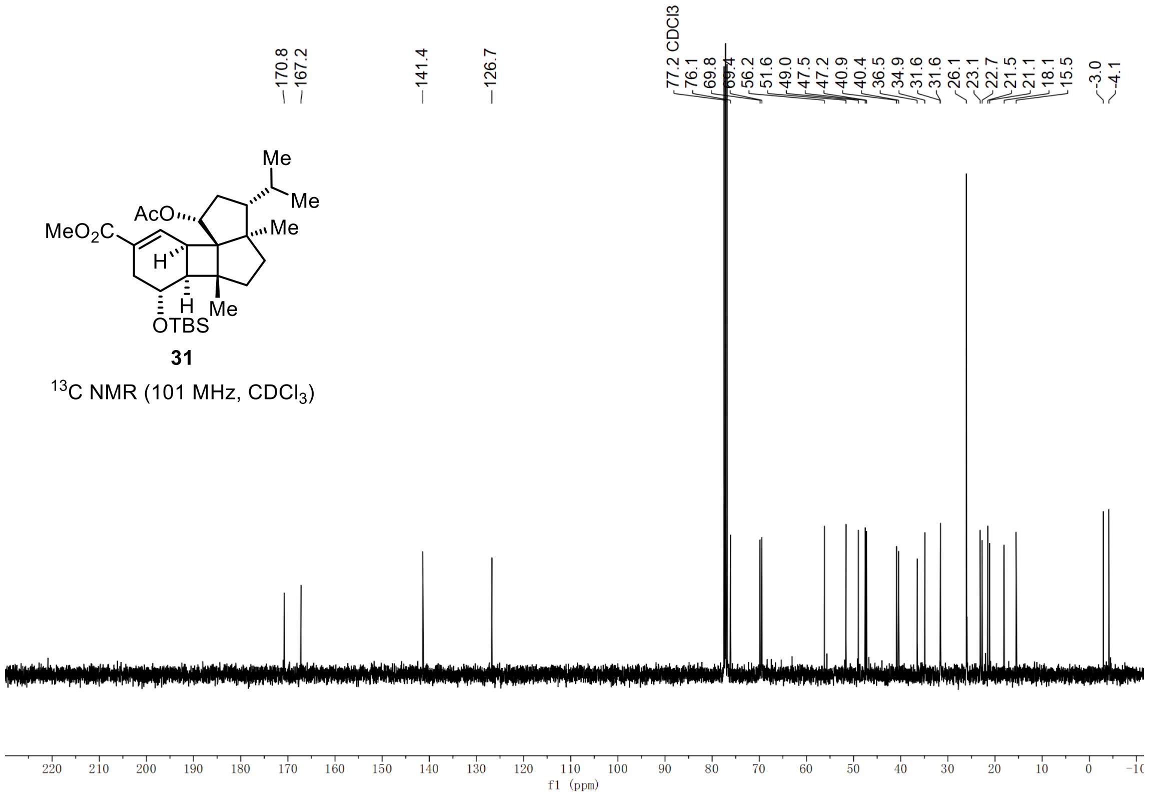

31

^{13}C NMR (101 MHz, $CDCl_3$)

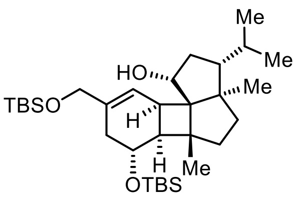

32

^{1}H NMR (400 MHz, $CDCl_3$)

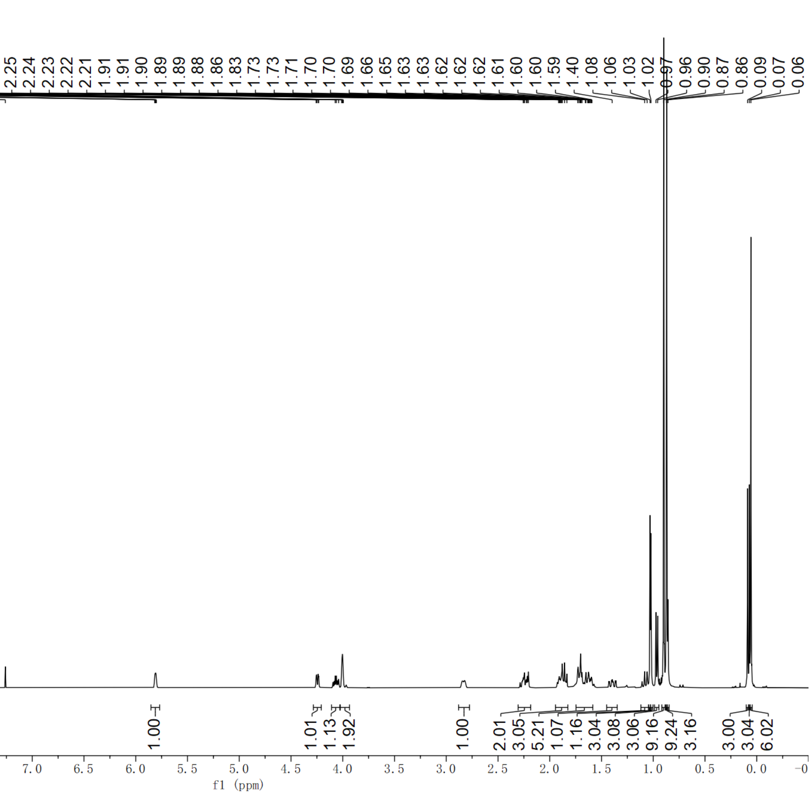

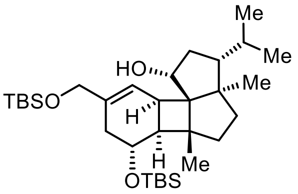

32

^{13}C NMR (101 MHz, $CDCl_3$)

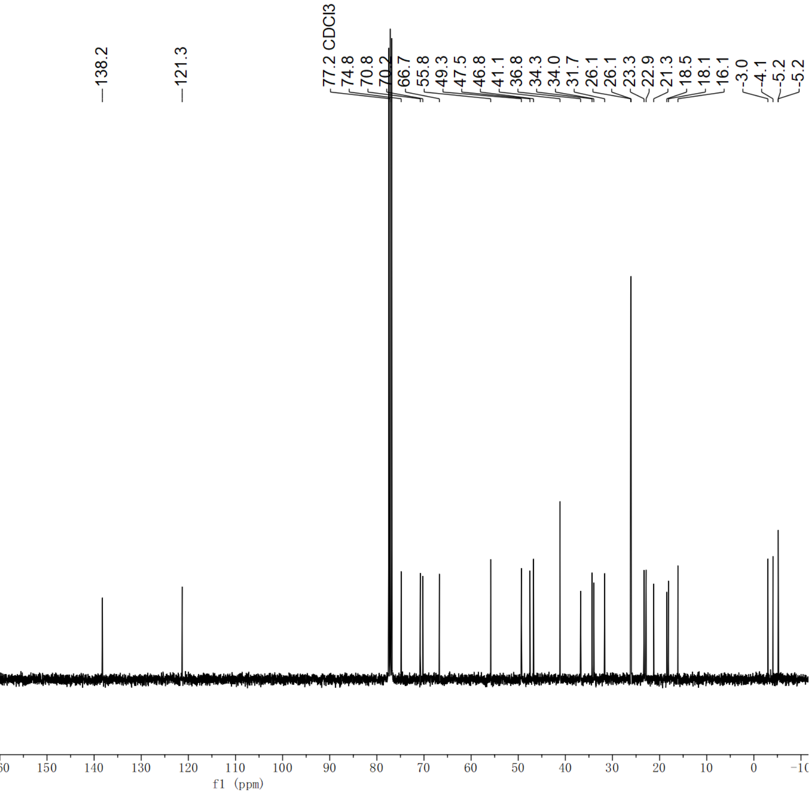

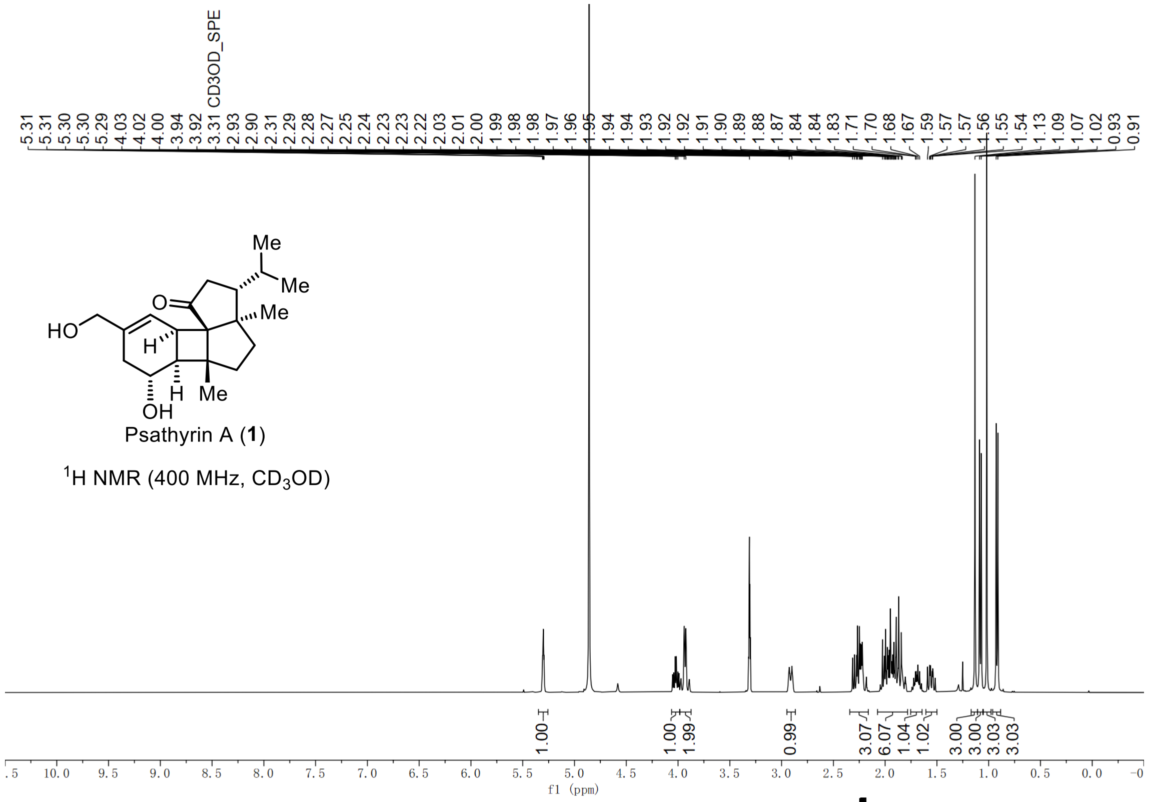

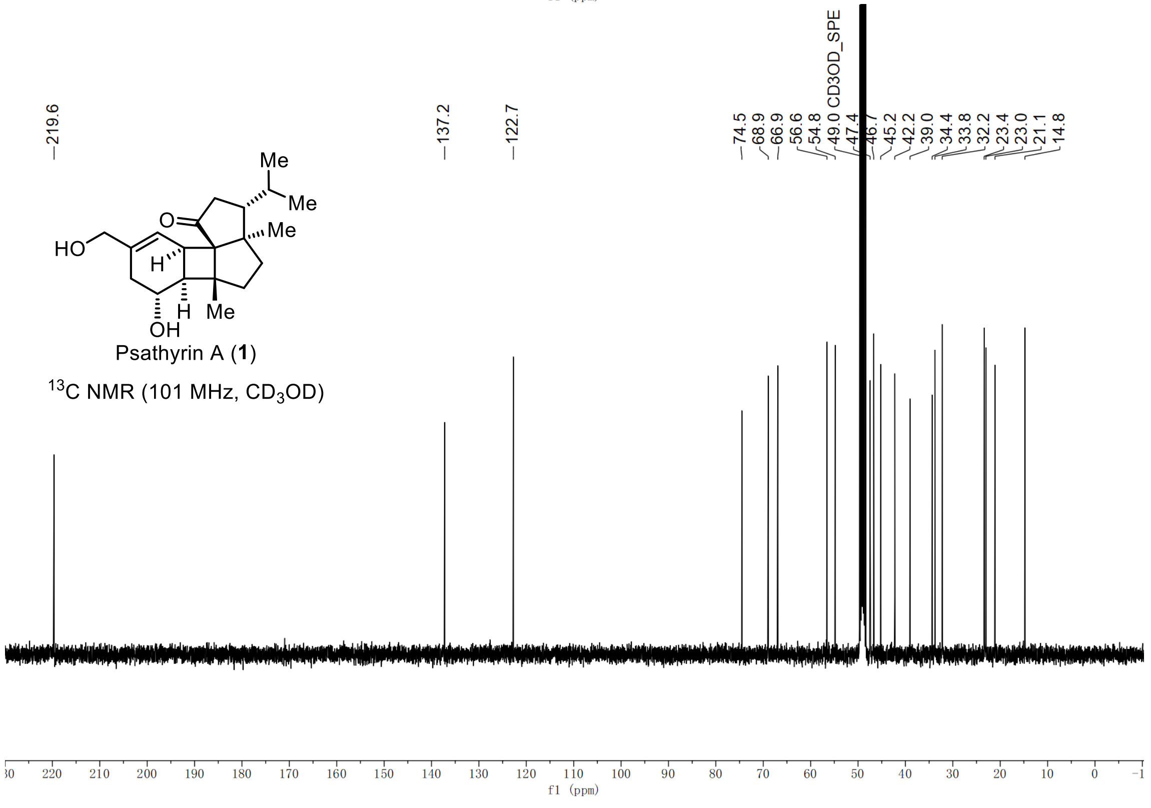

Supplement: Supplementary file 1 [file ja5c11534_si_001.pdf]
